# Silicified bulliform cells of Poaceae: morphological characteristics that distinguish subfamilies

**DOI:** 10.1186/s40529-020-0282-x

**Published:** 2020-03-02

**Authors:** Iju Chen, Kuang-ti Li, Cheng-hwa Tsang

**Affiliations:** Archaeology Department, Institute of History and Philology, Academia Sinica, No. 130, Section 2, Academia Rd., Nangang District, Taipei, 115 Taiwan

**Keywords:** Poaceae, Grasses, Bamboos, Phytolith morphotype, Silicified bulliform cells, Morphometric, Paleoenvironment, Archaeological, Sediments, Taiwan

## Abstract

**Background:**

Grass phytoliths are the most common phytoliths in sediments; recognizing grass phytolith types is important when using phytoliths as a tool to reconstruct paleoenvironments. Grass bulliform cells may be silicified to large size parallelepipedal or cuneiform shaped phytoliths, which were often regarded as of no taxonomic value. However, studies in eastern Asia had identified several forms of grass bulliform phytoliths, including rice bulliform phytolith, a phytolith type frequently used to track the history of rice domestication. Identification with a higher level of taxonomic resolution is possible, yet a systematic investigation on morphology of Poaceae bulliform phytoliths is lacking. We aimed at providing a morphological description of bulliform phytoliths of Poaceae from Taiwan based on morphometric measurements in anatomical aspect. The results are important references for paleo-ecological studies.

**Result:**

The morphology of grass bulliform phytoliths is usually consistent within a subfamily; the end profile is relatively rectangular in Panicoideae and Micrairoideae, whereas cuneiform to nearly circular in Oryzoideae, Bambusoideae, Arundinoideae, and Chloridoideae. Bulliform phytoliths were seldom observed in Pooideae. Certain morphotypes are limited to plants growing in specific environments. For example, large, thin, and pointed bulliform phytoliths are associated with wet habitat; Chloridoideae types are mostly from C4 plants occupying open arid places.

**Conclusion:**

Grass bulliform phytoliths can be identified at least to the subfamily level, and several forms were distinguished within large subfamilies. Previously un-reported silicified cell types, i.e., arm cells and fusoids, and two special trichome phytolith types associated with bulliform phytoliths, were described. Morphometric methods were great tools for delimiting morphotypes; with refined morphological classification the association between forms and habit/habitats was revealed. The knowledge provides new ways to interpret phytolith assemblage data, and it is especially useful when the sediments are enriched in large blocky phytoliths.

## Background

Phytoliths (plant opals, silica cells) are silica deposition in plants. The formation of phytoliths is specific: some plants/plant parts produce no phytolith at all, while others in abundance. Solid silica deposition usually fills up the whole cell lumen and the final product takes up the shape of the cell. Therefore, the taxonomic or anatomical origin of a phytolith may sometimes be recognized. Soon after the discovery in the 19th century, phytoliths had been considered a useful tool for environmental reconstruction, and the application in archaeological researches increased exponentially since the 1970s (Piperno 2006; Hart 2016).

Silica accumulation in leaves is a common characteristic of Poaceae. Grasses occupy a wide array of environments, and the dominant species often densely cover a large area, hence the ubiquity of grass phytoliths in ancient sediments. The silicified short cells of peculiar shapes, such as bilobates (dumbbells), saddles, and rondels, are present exclusively in the grass family. Although they are good representatives of Poaceae, usually there are more than one form of short cell phytoliths existing in one plant (multiplicity), and the same form can be found in more than one taxon (redundancy). Many works on grass short cell phytoliths have demonstrated how a careful assessment of the association between phytolith assemblages, taxonomy, and habitats is necessary before using phytoliths to reconstruct paleoenvironments (Lu and Liu 2003; Strömberg 2005; Barboni and Bremond 2009; Neumann et al. 2015). In addition to short cells, phytoliths of the grass family include those originated from long cells, trichomes, and bulliform cells. These phytoliths are larger than short cells, and are in general considered of limited value in discriminating taxa within the family.

While preparing sediments from archaeological sites in southern Taiwan, it was observed that large-size phytoliths were enriched, and short cell phytoliths frequently contained less than 1% in the phytolith assemblages (unpublished data). The reason could be that natural loss occurred more easily on small, fragile phytoliths (Feng et al. 2017; Cabanes and Shahack-Gross 2015). It was crucial to identify the large-size phytoliths while making ecological inferences from such sediments. Among the large phytoliths in fossil soils, those with parallelepipedal or cuneiform shape—likely originated from Poaceae bulliform cells—were the dominant types. In addition, a thorough phytolith survey on regional flora is necessary for paleoenvironmental reconstruction, yet only limited works (Chen 2009) had been carried out in Taiwan. Therefore, we were inspired to further investigate the morphology of bulliform phytoliths from grasses in Taiwan.

Several morphotypes of grass bulliform phytoliths had been recognized, mostly from paleoenvironmental studies in eastern Asia (Bowdery 1999; Lu et al. 2006; Miyabuchi and Sugiyama 2016; An et al. 2015). The described morphotypes included bulliform phytoliths of species in Panicoideae, Bambusoideae, Chloridoideae, and reeds (*Phragmites* sp.). The delimitation of these morphotypes was either not mentioned or based on very few extant species for comparison. In some studies, images of grass bulliform phytoliths were presented without description and classification (Motomura et al. 2010; Lu et al. 2002). On the other hand, morphology of rice bulliform phytoliths was scrutinized for use in study of rice domestication (Gu et al. 2013; Huan et al. 2015; Pearsall et al. 1995; Wang et al. 1996; Fujiwara 1993). Comparisons were usually made within Oryzoideae, assuming no occurrence of similar shape in other Poaceae subfamilies. It is clear that a more accurate association between morphotypes and specific environmental conditions can only be made from works with a large-scale sampling and careful systematic documentation. However, none of the current morphological studies on grass bulliform phytoliths have achieved the comprehensive levels as those on short cell phytoliths. Issues of multiplicity and redundancy as observed in short cell phytoliths have not yet been fully assessed for bulliform phytoliths.

Bulliform cells are enlarged leaf epidermal cells found in nearly all members of Poaceae and in most monocots. It was speculated that bulliform cells are involved in leaf rolling and expanding via regulating water intake, especially in occasions such as the unrolling of developing leaves during maturation (Metcalfe 1960; Ellis 1976). The genetic studies of leaf rolling in rice suggested that bulliform cell arrangement pattern is involved in this trait (Li et al. 2017b). Nevertheless, unequivocal evidence is still needed to fully understand the function of bulliform cells. Bulliform cells are not always silicified. Bulliform cells were found to be silicified in 8 out of 28 taxa (Kaufman et al. 1985). Silicification may depend on the presence of available silica in the soils, position of the leaf, and developmental status of the plant (Motomura et al. 2004; Sangster and Parry 1969; Honaine and Osterrieth 2012; Issaharou-Matchi et al. 2016; Li et al. 2017a; Liu et al. 2016; Dey et al. 2015). Silica uptake/deposition could be genetically and metabolically controlled, and in general it was believed that silica in plants is related to biotic and abiotic stresses (Ma and Yamaji 2006; Kumar et al. 2017). Whether silica deposition in bulliform cells carries further specific function is unknown, but the large cell size hints that they are possibly major water/silica reservoirs.

Since the size and shape of the adjacent cells and the arrangement pattern of bulliform cells decide the morphology of a bulliform phytolith, Poaceae leaf anatomy was consulted on a regular basis in this study. Leaf anatomy of major Poaceae tribes were described in selected studies (Metcalfe 1960; Renvoize 1982a, b, c, 1983, 1985a, b, c, 1986a, b, 1987; Wu 1962). The online images of leaf blade transverse (cross) section from “Gramineae in Flora of Taiwan” (Hsu et al. 2000) and “The grass genera of the world” (Watson et al. 1992) had provided easily accessible, valuable information.

Grass bulliform cells are located between veins (intercostal) on both epidermis, the upper epidermis only, or present only near upper mid-veins. Usually, identical bulliform cells stack neatly end to end along the axes of major veins forming a long pile. The facet toward leaf center is in contact with mesophyll cells or clear cells (enlarged colorless cells); therefore, when silicified, the bulliform phytolith carries the impression marks of those cells. The bulliform cells may or may not appear grouped to a pattern in a leaf cross section. When they are grouped, it is usually a pattern of sea shells or fans composed of three or more cells. The one located in the center, the median bulliform cell, is usually the largest and symmetrical bilaterally. Diagnostic features are most conspicuous on the median bulliform cell. The neighboring bulliform cells are usually relatively small, narrow in width, asymmetrical, and lacking typical features of a median bulliform cell. Phytoliths of non-median bulliform cells tend to position on their lateral sides and appear parallelepipedal, providing no taxonomical information.

It had long been recognized that leaf anatomy of Poaceae is useful for species identification. Patterns of bulliform cell arrangements in leaf cross section view—the frequency, distribution, and the relative size and shape of the cells—are of taxonomic importance (Ellis 1976, 1979; Metcalfe 1960). It is very likely that morphology of bulliform phytoliths carries diagnostic characters that distinguish phylogenetic groups. In spite of the importance, most of the previous reported grass bulliform phytolith types were not described in the context of leaf anatomy. Therefore, in this study, we focused on observing morphology of bulliform phytoliths based on the associated anatomical features. The plant materials were common grasses from various habitats in Taiwan, in hope to provide a basic framework for further local paleoenvironmental studies. Shape variations usually brought up the issue of morphotype delimitation; hence morphometric methods were applied. Based on the analyses, morphological characteristics shared by related groups were identified, and the association between morphology and growing environment were discussed.

## Methods

### Plant collection

Common grasses from various habitats throughout the island Taiwan were collected for phytolith extraction (Table 1). Dominant species that occupy a large area and reoccur at the same place every growing season are the main targets of this study. With the Tropic of Cancer passing through the middle, Taiwan is in subtropical climate zone. Nevertheless the island is mountainous, with a great altitude fluctuation between 1000 and 3000 m in 30% of its area; therefore vegetation such as temperate montane forest and alpine tundra also exist (Hsieh and Shen 1994). Typical habitats of Poaceae in low-altitude regions are categorized as follows:

**Table 1:**
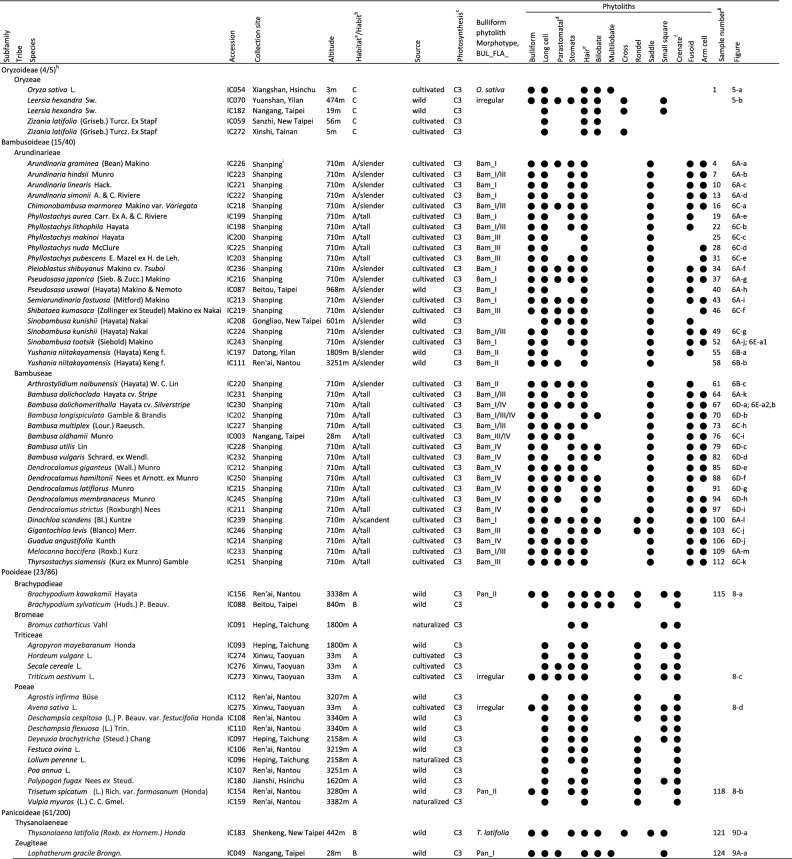

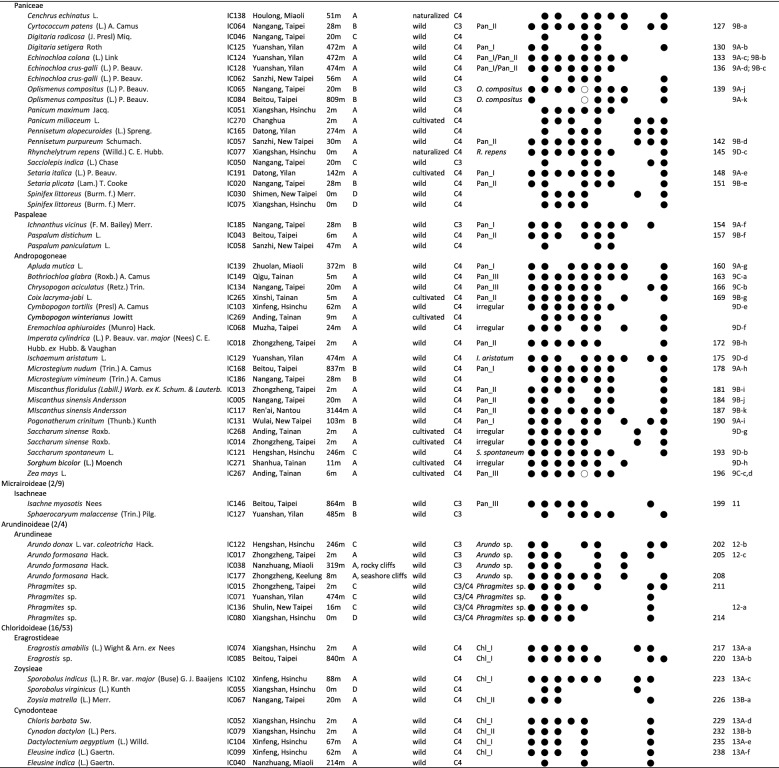
Poaceae species sampled for leaf phytoliths in this study

^a^Habitat of collection site. A, Sunny; B, Shady to partial shady; C, Near water; D, Salt adapted. See “Methods”

^b^Bambusoideae, slender, scandent, or tall. See text

^c^Genus-level, data from Osborne et al. (2014) and Zheng et al. (2000)

^d^Parastomatal long cells, usually with both ends deeply concave

^e^White circle indicates presence of special hair type

^f^Tablet with crenate margin, or Pooideae wavy trapezoid

^g^The sample number in Additional file 1: Appendix S1. The first of the 3 constitutive number for each measured accession is shown in this column

^h^Genus/species numbers described in Flora of Taiwan (Hsu et al. 2000); Phyllogeny is based on Soreng et al. (2015)

^i^Shan-Ping Forest Ecological Garden, Lioukuei Research Center, Taiwan Forestry Research Institute

A. Sunny: including open grasslands, exposed hill slopes, rocky cliffs.
B. Shady to partial shady: forest understory, forest edges.
C. Near water: pond sides, along a stream, on exposed riverbeds, paddy fields; sunny.
D. Salt adapted: river mouths, salt marshes, sandy beaches.

Above 1500 m, Poaceae can be found on exposed open lands, rocky slopes (A), or understory of evergreen forests (B).

A brief descriptions for each grass subfamily in Taiwan (Hsu et al. 2000) was given bellow:

Oryzoideae: most species in this subfamily are emergent. *Oryza sativa* and *Z. latifolia* are widely cultivated on the island
Bambusoideae: tall bamboos are widely cultivated. In northern Taiwan, open hillsides on mountains 500–1000 m high are commonly occupied by shrubby slender bamboos *P. usawai* or *S. kunishii*. Dwarf form of *Y. niitakayamensis* covers large area of exposed land surface on mountains above 3000 m high. This species is also very wide-spread in shady conifer forests above altitude of 2000 m and can grow up to 2 m high
Pooideae: they are dominant taxa in mountains above 2000 m high, with the exceptions of *P. annua* and *P. fugax*, both are also very common in low lands
Panicoideae: tall grasses *Miscanthus* spp. are common on the sunny hillsides. *S. spontaneum* occupies every exposed river beds while in seasons. Tall reed-like plants *T. latifolia* are found in forest edges. *S. littoreus* frequently covers large areas on sandy beaches. In low altitude regions, many common short grasses found in sunny spots or shady forests belong to Panicoideae. *M. sinensis* is wide spread in both lowlands and alpine regions. The alpine plants are dwarf with smaller inflorescences comparing to those from lowlands
Micraioideae: they are mostly distributed in wet, exposed or partial shady areas
Arundinoideae: *Arundo donax* and *Phragmites* sp. are cosmopolitan tall reeds frequently growing on edge of water. *A. formosana*, endemic to Taiwan and parts of Ryujyu Islands, are usually found hanging on rocky cliffs in low-altitude mountains, river banks, or dry seashore cliffs (where the plants are specialized to dwarf form)
Chloridoideae: species in this group are mostly C4 plants in arid places. *C. barbata* and *E. indica* are weedy species in dry open lands. *S. virginicus* usually occupies large areas on river mouth salt marshes. *Z. matrella* are common in seashore grassland

Major Poaceae crop plants were collected in open fields. Bamboo leaves were mainly collected in Shan-Ping Forest Ecological Garden, Lioukuei Research Center, Taiwan Forestry Research Institute. Only plants with inflorescences were collected (except for bamboos) to ensure proper identifications. Species level taxonomy was based on Flora of Taiwan (Hsu et al. 2000). Among the bamboos sampled in this study, *A. naibunensis* is treated as *Ampelocalamus naibunensis* (Hayata) T. H. Wen, *S. kunishii* is placed under *Gelidocalamus*, and species of *Arundinaria* are placed under genus *Pleioblastus* or *Pseudosasa* in Flora of China (Wu et al. 1994). Classification and general information of Poaceae worldwide were based on Soreng et al. (2015). Genus-level photosynthetic types were based on Osborne et al. (2014); and those of *Phragmites* on Zheng et al. (2000). For the same species, only one accession was extracted for phytoliths. In the case where bulliform phytoliths were scarce, additional accession was processed if available. Accessions from different habitats were observed for species with broad habitat ranges. Plants were air-dried and deposited in the Archaeology department, Institute of History and Philology, Academia Sinica.

### Phytolith extraction

Phytoliths were prepared by the standard acid extraction method (Pearsall 2000; Piperno 2006; Jenkins 2009). Dry ashing preparation usually results in phytoliths conjoined to large sheets/piles while wet oxidation obtains mostly separate single grains (Jenkins 2009). Since the purpose of this study is to document detailed morphology of a single grain, acid extraction method was used. Only mature leaves were processed since silica deposition may not be completed in young leaves (Parry and Smithson 1958). The whole mature leaves were first cut into pieces of 2–3 cm in length and placed in 50 ml autoclavable polypropylene centrifuge tubes. Leaves were soaked in 1% detergent for at least 4 h and sonicated for 20 min. After sonication, leaves were placed in a Büchner funnel, cleaned by running water, and dried in an oven of 50° overnight. Prepared leaves were cut into 0.5 cm long pieces, and 0.2 g of these leaf pieces were placed in clean glass tubes for acid treatment. Five milliliters of nitric acid was added into each tube, and the tubes were placed in a water bath at nearly boiling temperature. Dashes of potassium chlorate were slowly added into the tubes to speed up the reaction. Usually the reaction completed after 1.5 to 2 h of treatment. The acid solution was carefully removed to 50 ml polypropylene tubes. The phytoliths were washed with distilled water by centrifugation 3 times. Water was removed and phytoliths were soaked in 5 ml 10% hydrogen chloride for 5 min. The phytoliths were washed again with distilled water by centrifugation 3 times. Prepared phytoliths were moved to an Eppendorf, dried in a 50° oven overnight, and kept in a desiccator. Phytoliths were mounted in 50% glycerol and observed under a microscope (Leica DM2500 P) of 400× magnification. Phytoliths were turned around by gentle touches on the cover slip with a sharp pointed tool. Images of the same grain in different orientations were recorded with a Nikon D5100 camera.

### Phytoliths measurements and statistical tests

**Fig. 1:**
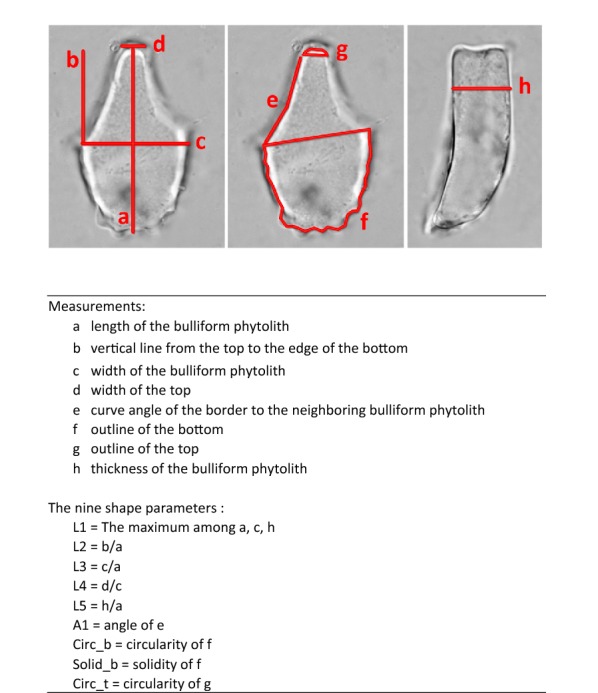
Measurements and the nine shape parameters of Poaceae bulliform phytoliths. A grain of bulliform phytolith from *B. oldhamii* is shown as an example. **a**–**g** end view, **h** lateral view

For each accession, at least 50 bulliform phytoliths were observed, and 3 grains with the most frequently observed shape were measured as in Fig. 1. The original measurements were listed in Additional file 1: Appendix S1. Software ImageJ (Rasband 1997–2016) was used for the measurements. The implemented measuring methods in ImageJ, “Shape Descriptors”, including “circularity” and “solidity”, were used in this study. The morphotypes were recognized by either of the two criteria: (1) arbitrarily delimited by clustering method with nine shape parameters; (2) visually distinct from closely related taxa. Principle component analysis (PCA; unit variance scaling is applied; SVD with imputation is used to calculate principal components) and cluster analysis (unit variance scaling is applied; distance measured by correlation, average linkage criteria) were performed via a web tool ClustVis (Metsalu and Vilo 2015). The bean plots were prepared by using BoxPlotR (Spitzer et al. 2014).

### Terminology related to morphology of bulliform phytoliths

Unless otherwise indicated, the morphological descriptions/measurements in this article apply to the median bulliform phytoliths only. In a leaf transverse section, median bulliform phytoliths are more or less flabellate in shape and suitable to the ICPN 2.0 morphotype name BULLIFORM_FLABELLATE (International Committee for Phytolith Taxonomy 2019). Code BUL_FLA was applied as prefix to all morphotype names designated in this article but not shown to save space.

Since bulliform cells typically occur on leaf adaxial surface, bulliform phytoliths were described as their orientation on leaf adaxial surface when the adaxial surface was positioned upward (Fig. 1). The same terms for describing the orientation of a bulliform phytolith in Motomura et al. (2010) were applied in this article. In the 3-dimensional aspect, end view/end profile refers to the facet revealed in leaf cross section; side view/lateral refers to the side revealed in leaf parallel-vein longitudinal section; top refers to the facet exposed on the leaf adaxial surface; bottom refers to the part adjacent to mesophyll tissues/clear cells. The thickness of a bulliform phytolith refers to the horizontal distance in lateral view, and the length and the width of a bulliform phytolith refer to those on an end profile. To avoid confusion, “the lateral side of end profile” (Motomura et al. 2010) was described as “neighboring border” or “border to the neighboring bulliform cell/phytolith” in this article.

## Results

### Types of silicified cells in leaves of Poaceae

Common grasses from native habitats in Taiwan, cultivated crop plants, and bamboos from a specimen garden, were collected for this study. A total of 110 species in 7 subfamilies were studied for their bulliform phytoliths (Table 1). It was observed that almost every leaf cell type can be silicified. Silicified non-epidermal tissues, such as tracheary elements, mesophylls, and vascular parenchyma, were present in some samples. Usually epidermal tissues were heavily silicified. Trichomes, including prickles, macrohairs, and microhairs, were frequently silicified. Short cell phytoliths were present in all accessions, whereas the silicification of other epidermal cell types varied. Bulliform phytoliths usually existed in a large quantity if present. The presence of silicified cell types, along with the locality and habitat information of the observed taxa, were listed in Table 1.

### Dissecting shape of a bulliform phytolith

Minor variations in shape are common among bulliform phytoliths from the same plant, and mathematical measurement is one of the best ways to describe the continuous variations. Nine geometric parameters were set up to represent the shape of a bulliform phytolith in an anatomical context (Fig. 1). L1 represents the size of the phytolith, L2 represents the relative length difference to the neighboring bulliform phytoliths, L3 indicates the width to length ratio of the end profile, L4 shows the relative width of the top in end view, L5 indicates the relative thickness of the phytolith. A1 indicates the curve degree of the border to the neighboring bulliform phytolith. Circ_b represents the circularity of the bottom. Solid_b represents the roughness of the bottom. Circ_t indicates convex degree of the top. Some of the length measurements were similar to those from the previous morphometric studies of rice bulliform phytoliths (Fujiwara 1993; Gu et al. 2013). The parameters measuring the curvy parts of the bulliform phytoliths had not been used before. It was observed that these curvedness-related parameters vary significantly among phylogenetically distant taxa. They might increase the grouping resolution and were therefore included in the morphometric analysis. One great advantage of defining the morphometric characters in an anatomical context is that the information can be easily used in taxonomic works, the studies of character evolution, or researches related to bulliform cells.

### Grass bulliform phytoliths can be distinguished at subfamily level

**Fig. 2:**
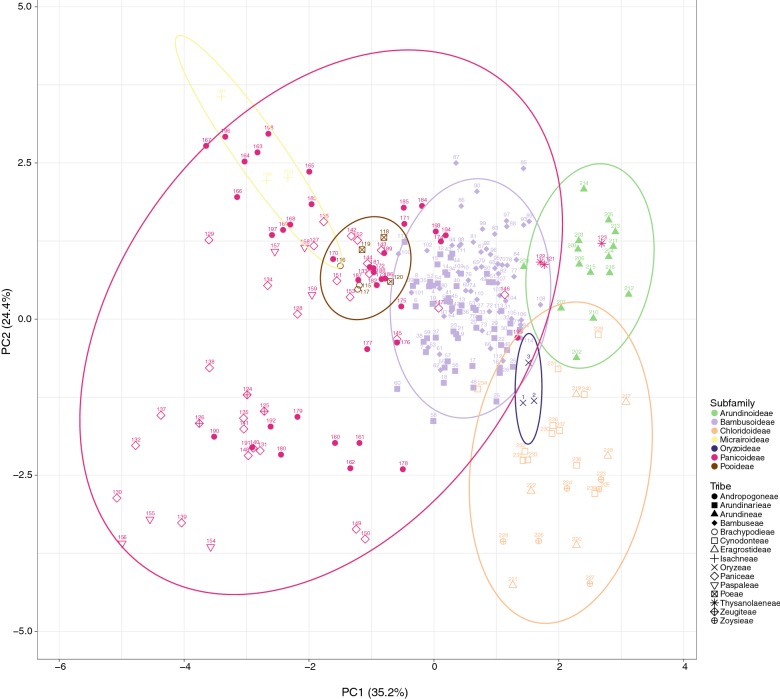
PCA of Poaceae bullilform phytoliths with nine shape parameters. X and Y axis show principal component 1 and principal component 2 that explain 35.2% and 24.4% of the total variance, respectively. Prediction ellipses are such that with probability 0.95, a new observation from the same group will fall inside the ellipse. N = 240. Each data point is labeled with the sample number in Table 1, Additional file 1: Appendix S1

**Fig. 3:**
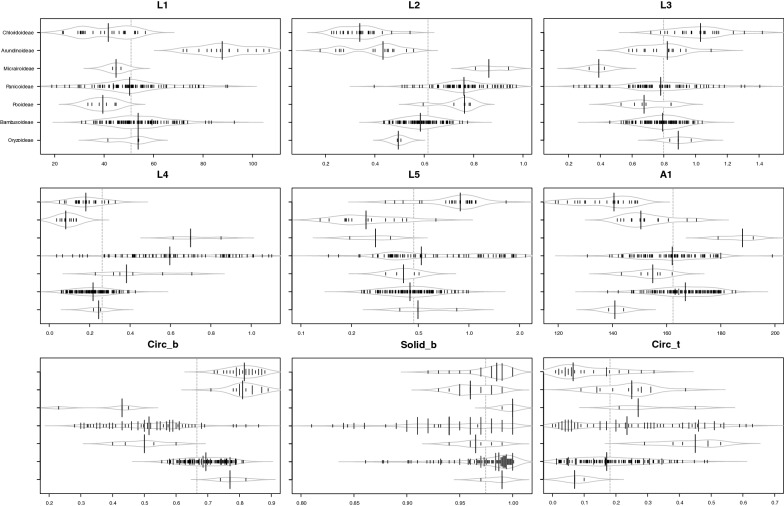
Bean plots of the nine shape parameters, showing the variation of bulliform phytoliths among the Poaceae subfamilies. Long lines show the medians; short lines represent individual data points; areas represent the estimated density of the data. Subfamilies are arranged in the same order in all graphs

PCA was performed to assess the shape similarity of grass bulliform phytoliths (Fig. 2). Bulliform phytoliths of subfamilies Oryzoideae, Bambusoideae, Arundinoideae, and Chloridoideae formed their own groups in ordination space. Those of Panicoideae show great variations and are not well separated from Pooideae and Micrairoideae. Within each subfamily, distinct clusters of tribes are not present. L2, L4, and Circ_b contribute to principle component 1 greatly, and L3 and L5 weight most in principle component 2 (Table 2). As the PCA loadings suggested, relative size difference between median and neighboring bulliform cells (L2), whether the top is narrow or wide (L4), the shape of bottom (Circ_b), width to length ratio in end view (L3), and the relative thickness (L5) are important for distinguishing subfamilies. Beanplots of the nine shape parameters show the variation ranges of each subfamilies (Fig. 3). End profiles of bulliform phytoliths of Panicoideae, Pooideae, and Micrairoideae are relatively rectangular because of larger L2, larger L4 and smaller Circ_b (Fig. 3); whereas those of Oryzoideae, Bambusoideae, Arundinoideae, and Chloridoideae are cuneiform with obvious circular bottoms (small L2, small L4, and large Circ_b).

**Table 2:** Component loadings of PCA in Fig. 2

	PC1	PC2	PC3	PC4	PC5	PC6	PC7	PC8	PC9
L1	0.17362	− 0.35715	− 0.15637	0.71268	0.24118	− 0.47388	0.14771	− 0.02886	0.0675
L2	− 0.48022	− 0.23344	0.10928	− 0.15278	0.09688	− 0.17102	0.41458	0.59926	− 0.33165
L3	0.11821	0.56735	0.07117	− 0.08668	0.46235	− 0.13102	0.55851	− 0.29704	− 0.14306
L4	− 0.5065	0.08729	− 0.01	0.06201	− 0.26364	− 0.39232	− 0.22899	− 0.51009	− 0.44255
L5	− 0.24142	0.53219	0.01392	0.13746	0.28997	− 0.28057	− 0.49253	0.40585	0.26326
A1	− 0.19402	− 0.26999	0.61566	0.13561	0.52805	0.33201	− 0.23162	− 0.20951	− 0.07367
Circ_b	0.53435	0.0779	0.03238	0.02319	0.06566	− 0.02209	− 0.30977	0.24744	− 0.73813
Solid_b	0.2933	− 0.16885	0.51826	− 0.42793	− 0.09447	− 0.60955	− 0.04966	− 0.03549	0.22258
Circ_t	− 0.03532	− 0.31265	− 0.55639	− 0.48625	0.52251	− 0.11883	− 0.21744	− 0.14335	− 0.00501

**Fig. 4:**
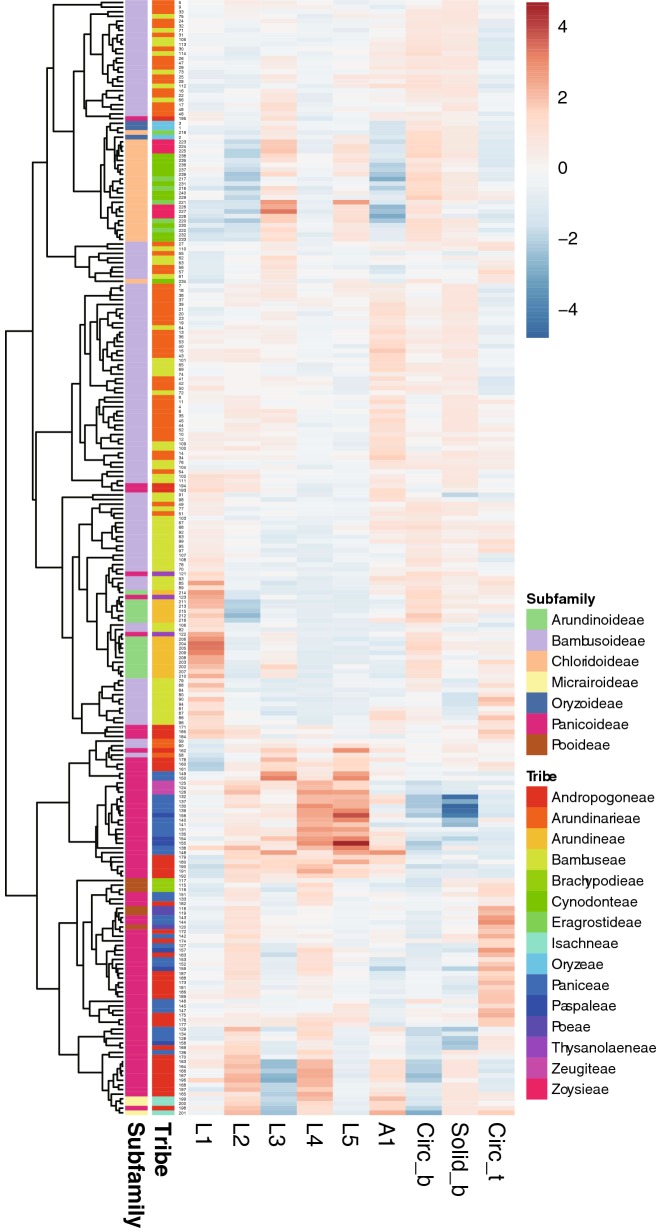
Cluster diagram and heat map of shape parameters from the analysis including all measured samples in Poaceae. The numbers are identical to the sample numbers in Table 1

Alternatively, morphological similarity of bulliform phytoliths was tested by clustering method (Fig. 4). Subfamilies are mostly congregated in the same clusters. Heatmaps of shape parameters clearly show that L2, L4, and Circ_b are the main contributors for the separation of the two major groups: rectangular-like (Panicoideae, Pooideae, and Micrairoideae) and cuneiform-like (Oryzoideae, Bambusoideae, Arundinoideae, and Chloridoideae). Most tribes were not forming groups in the cluster analysis. The results of clustering analysis were similar to those of PCA.

### Shared morphological characteristics and variation within a subfamily

#### Subfamily Oryzoideae

**Fig. 5:**
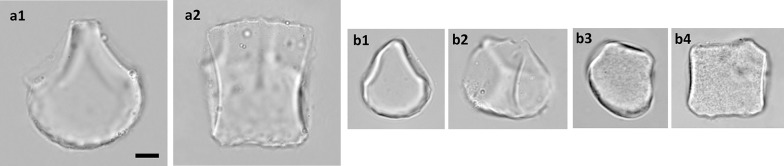
Bulliform phytoliths of subfamily Oryzoideae. **a***O. sativa*, **b***L. hexandra*. **1**, **3**, end view; **2**, **4**, lateral view. Bar 10 μm, all pictures are in the same scale

Typical bulliform phytoliths of *O. sativa* have a large round bottom and a narrow top, the surface of the bottom has shallow scale-like indentations (Fig. 5-a). The neighboring bulliform cells are usually half the size of the median bulliform cells (L2 around 0.5, Fig. 3), and the neighboring borders are curved slightly (A1 around 140, Fig. 3). The top of the bulliform phytolith is usually flat and not convex (Round_t small, Figs. 3, 5-a1). The thickness is usually less than the length (L5 < 1, Fig. 3). Comparing to those of *O. sativa*, bulliform phytoliths of *L. hexandra* (Fig. 5-b) are less abundant, smaller in size, highly various in shape, and their top sides are usually asymmetrical (Figs. 3, 5-b1). Bulliform phytoliths were not observed from *Z. latifolia* in this study. Scooped bilobate phytoliths were typical of this subfamily, which were also observed in all sampled Oryzoideae.

#### Subfamily Bambusoideae

**Fig. 6:**
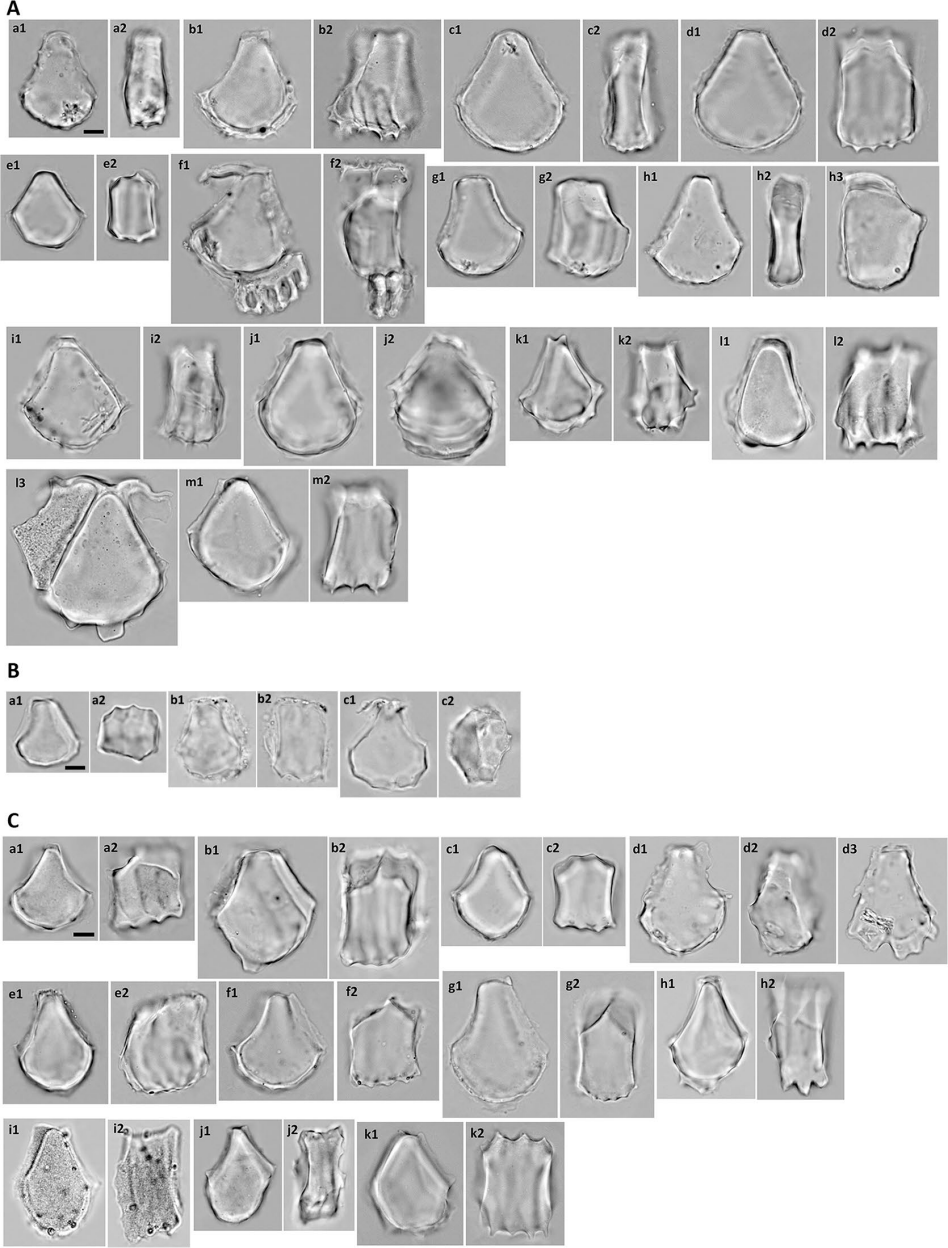

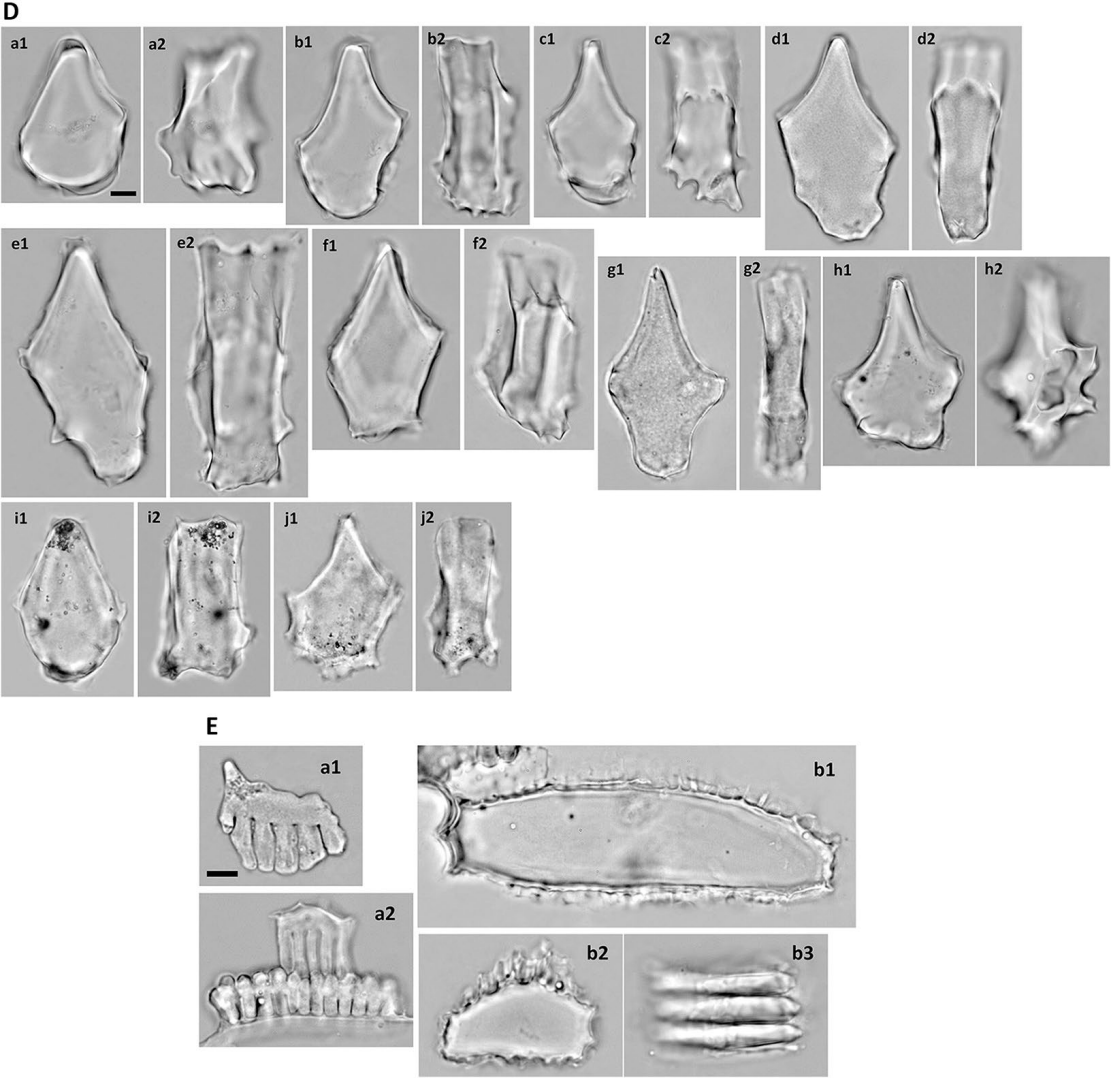
**A** Bulliform phytoliths of subfamily Bambusoideae, morphotype Bam_I. **a***A. graminea*, **b***A. hindsii*, **c***A. linearis*, **d***A. simonii*, **e***P. aurea*, **f***P. shibuyanus*, **g***P. japonica*, **h***P. usawai*, **i***S. fastuosa*, **j***S. tootsik*, **k***B. dolichochlda*, **l***D. scandens*, **m***M. baccifera*. **1**, **h3**, **l3**, end view; **2**, lateral view; **j2**, end view tilted to show ridges on the bottom. **f** Two silicified arm cells attached, **l3** a silicified neighboring bulliform cell attached. Bar 10 μm, all pictures are in the same scale. **B** Bulliform phytoliths of subfamily Bambusoideae, morphotype Bam_II. **a**, **b***Y. niitakayamensis*; **c***A. naibunensis*. **a** IC197; **b** IC111. **1**, end view; **b2**, lateral view; **a2**, **c2**, bottom view. Bar 10 μm, all pictures are in the same scale. **C** Bulliform phytoliths of subfamily Bambusoideae, morphotype Bam_III. **a***C. marmorea*; **b***P. lithophila*; **c***P. makinoi*; **d***P. nuda*; **e***P. pubescens*; **f***S. kumasaca*; **g***S. kunishii*; **h***B. multiplex*; **i***B. oldhamii*; **j***G. levis*; k, *T. siamensis*. **1**, end view; **2**, lateral view. Bar 10 μm, all pictures are in the same scale. **D** Bulliform phytoliths of subfamily Bambusoideae, morphotype Bam_IV. **a***B. dolichomerithalla*; **b***B. longispiculata*; **c***B. utilis*; **d***B. vulgaris*; **e***D. giganteus*; **f***D. hamiltonii*; **g***D. latiflorus*; **h***D. membranaceus*; **i***D. strictus*; **j***G. angustifolia*. **1**, end view; **2**, lateral view. Bar 10 μm, all pictures are in the same scale. **E** Phytoliths of subfamily Bambusoideae. **a** arm cells; **b** fusoids. **a1***S. tootsik*; **a2**, **b***B. dolichomerithalla*. **a**, **b1**, **b2** end view; **b3** lateral view. **b3** three fusoids attached. Bar 10 μm, all pictures are in the same scale

The bulliform phytoliths of Bambusoideae are cuneiform, with round bottoms and narrow tops (Fig. 6). The length is usually longer than the width (L3 around 0.8, Fig. 3). The bottom is frequently irregularly wavy or angular, a characteristic unique to this subfamily (Fig. 6D). The neighboring bulliform cells are smaller than the median ones (L2 0.4–0.8, Fig. 3), and the neighboring borders are straight to slightly curved (A1 140–180, Fig. 3). Besides the geometric characters, bulliform phytoliths of Bambusoideae have a unique feature with the surface of the bottom carries two to several parallel ridges which is hard to described by the shape parameters (lateral view, Fig. 6). The ridges are usually prominent and irregularly wavy. The grooves between the ridges are the impressions of the arm cells (flat mesophyll cells with digitation on abaxial side only, or around the edge; Fig. 6A-f, E-a). This character is present in all observed Bambusoideae except for *Y. niitakayamensis* and *A. naibunensis* (Fig. 6B). In these two species, regular mesophyll cells instead of arm cells leave the usual round scale-like indentations on the bottoms of their bulliform phytoliths. Fusoids, flat large irregular oval clear cells in mesophyll tissues, are another anatomical feature common in Bambusoideae. Silicified arm cells and fusoids were frequently observed in bamboo leaves but not present in other subfamilies (Table 1; Fig. 6E).

**Fig. 7:**
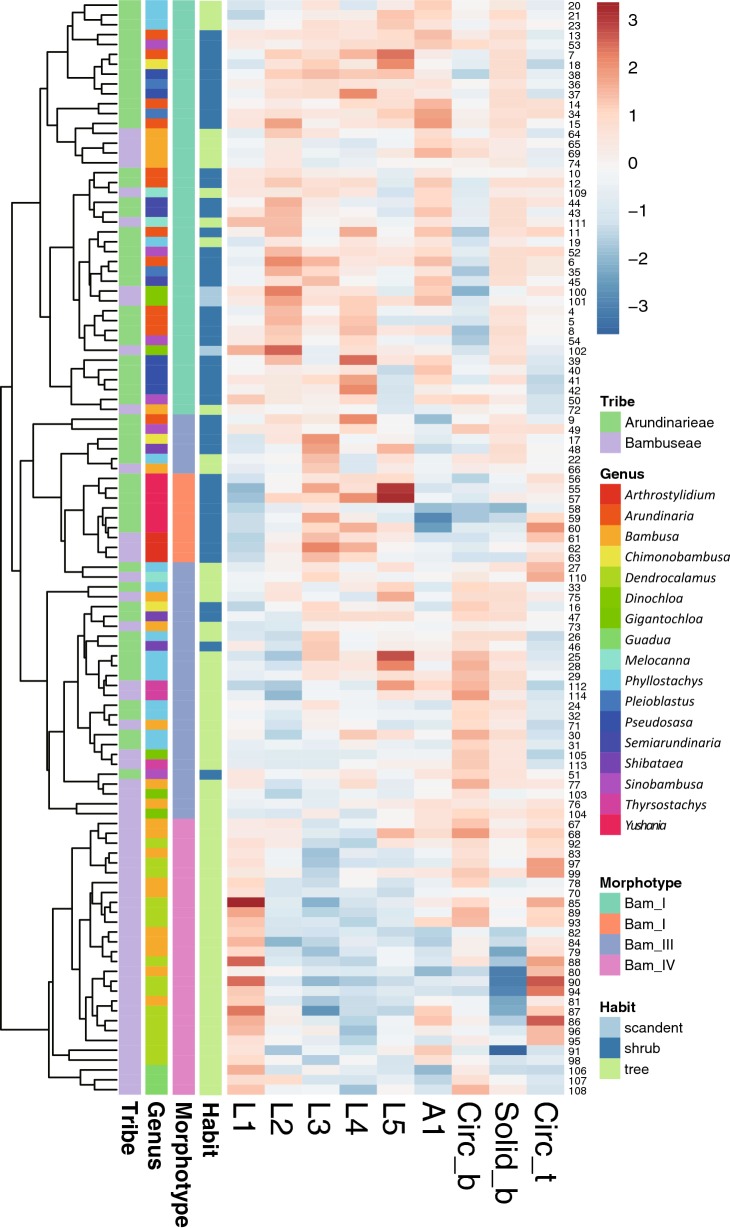
Cluster diagram and heat map of shape parameters from the analysis including measured samples of subfamily Bambusoideae. The numbers are identical to the sample numbers in Table 1

The morphology of bulliform phytoliths of Bambusoideae were summarized into four major forms by clustering method (Fig. 7). Bam_I is defined by long and straight neighboring borders, wide tops, and shallow bottoms (Fig. 6A; larger L2, larger A1, smaller Circ_b, Fig. 7). Type Bam_II are small, fan-shaped, with arched neighboring border, and—most importantly—with the scale-like indentations instead of parallel ridges on the surface of the bottoms (Fig. 6B; small A1, Fig. 7). Bam_IV is visually distinct by having an elongate outline with length longer than width, a sharp pointed top, and a prominent irregular wavy bottom (Fig. 6D; smaller L3, L4, Solid_b, Fig. 7). Type Bam_III represent the intermediate/hybrid forms of Bam_I and Bam_IV (Fig. 6C).

In Bambusoideae, morphotypes specific to a single species are lacking, and different types sometimes exist in the same plant (Table 1, Fig. 7). However, most species of *Pseudosasa* and *Pleioblastus* have type Bam_I bulliform phytoliths, *Y. niitakayamensis* and *A. naibunensis* have type Bam_II bulliform phytoliths, *Phyllostachys* typically have type Bam_III, and type Bam_IV are found only in Tribe Bambuseae (species of *Bambusa* and *Dendrocalamus*). Interestingly, shape of bulliform phytoliths is roughly correlated with growth habit (Fig. 7). Shrubby or dwarf bamboos with slender stems (less than 2 cm in diameter of full-grown stem) mostly produce Bam_I or Bam_II bulliform phytoliths. Large bamboos with thick stems reaching more than 2 m tall tend to have type Bam_III or Bam_IV bulliform phytoliths.

Bulliform phytoliths from the two different growth forms of *Y. niitakayamensis* are indistinguishable (Fig. 6B-a, b). Almost all sampled bamboo leaves contain great amounts of bulliform phytoliths. This feature is unusual comparing to other subfamilies of Poaceae (Table 1).

#### Subfamily Pooideae

**Fig. 8:**
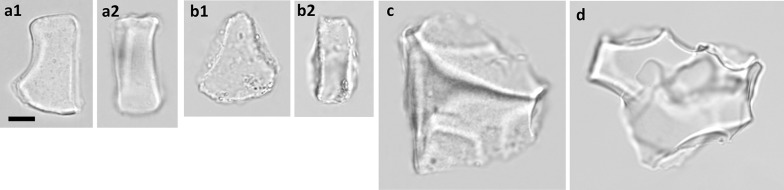
Bulliform phytoliths of subfamily Pooideae. **a***B. kawakamii*; **b***T. spicatum* var. *formosanum*; **c***T. aestivum*; **d***A. sativa*. **1**, end view; **2**, lateral view. Bar 10 μm, all pictures are in the same scale

Bulliform phytoliths were observed only in *B. kawakamii* (Fig. 8-a) and *T. spicatum* var. *formosanum* (Fig. 8-b), and they are similar to the morphotype Pan_II (see section below), indicated by PCA and cluster analysis (Figs. 2 and 4). A small amount of large, irregular-shaped phytoliths were observed in *T. aestivum* (Fig. 8-c) and *A. sativa* (Fig. 8-d). It is uncertain whether they are originated from bulliform cells. The rectangular, crenate margined Pooideae-specific phytoliths exist in all sampled taxa.

#### Subfamily Panicoideae

**Fig. 9:**
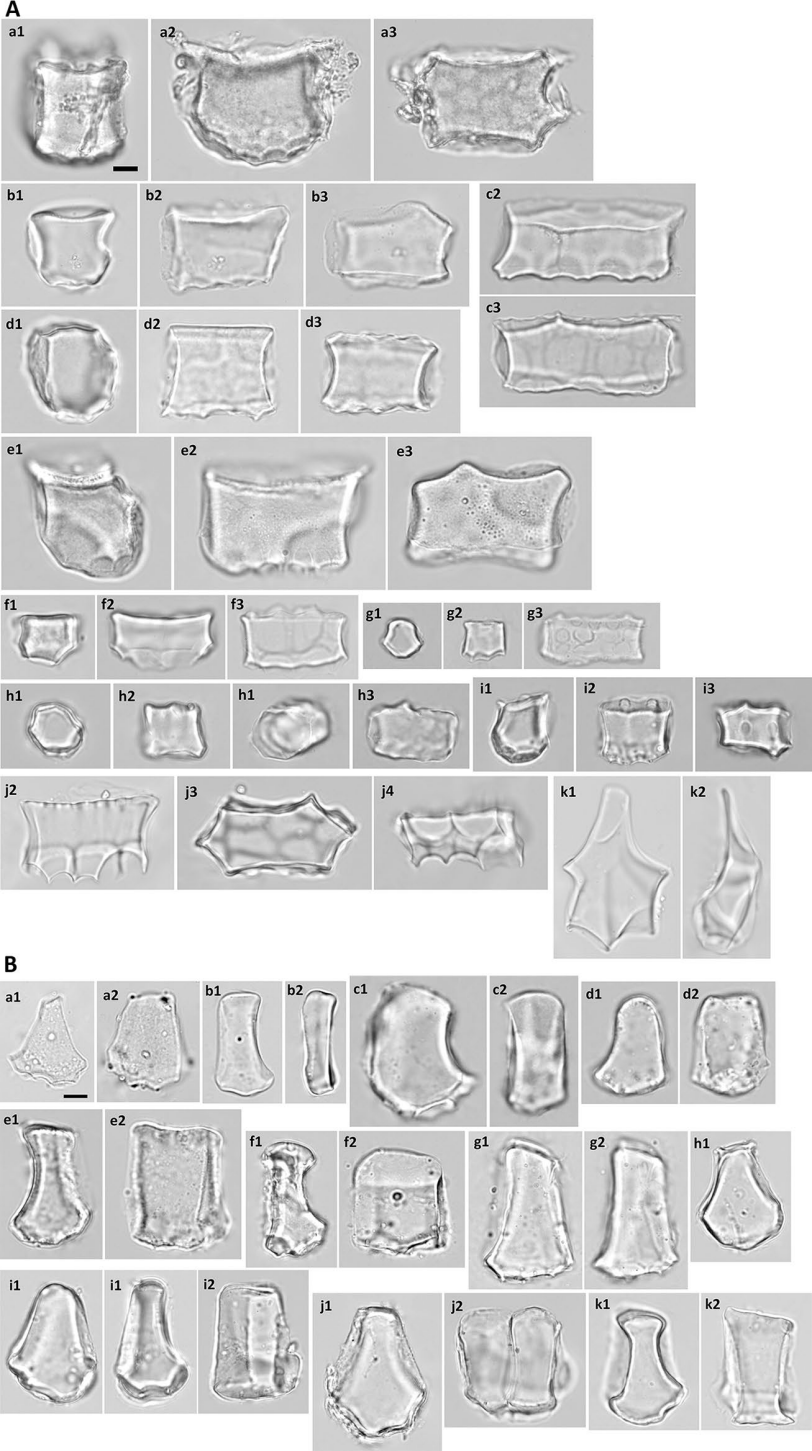

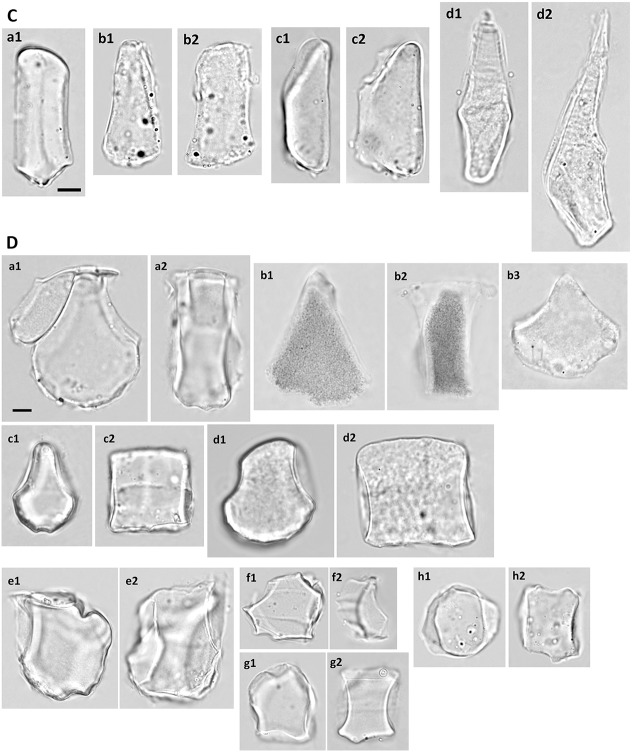
**A** Bulliform and associated hair phytoliths of subfamily Panicoideae, morphotype Pan_I. **a**–**j**, bulliform phytoliths; **k** hair phytoliths. **a***L. gracile*; **b***D. setigera*; **c***E. colona*; **d***E. crus*-*galli*; **e***S. italic*; **f***I. vicinus*; **g***A. mutica*; **h***M. nudum*; **i***P. crinitum*; **j**, **k***O. compositus*. **1**, end view; **2**, lateral view; **3**, top view; **4**, bottom view. Bar 10 μm, all pictures are in the same scale. **B** Bulliform phytoliths of subfamily Panicoideae, morphotype Pan_II. **a***C. patens*; **b***E. colona*; **c***E. crus*-*galli*; **d***P. purpureum*; **e***S. plicata*; **f***P. distichum*; **g***C. lacryma*-*jobi*; **h***I. cylindrica* var. *major*; **i***M. floridulus*; **j**, **k***M. sinensis*. **j** IC005; **k** IC117. **1**, end view; **2**, lateral view. **g2**, **i2**, turned slightly to show both sides. **j2**, two grains attached. Bar 10 μm, all pictures are in the same scale. **C** Bulliform and associated hair phytoliths of subfamily Panicoideae, morphotype Pan_III. **a**–**c** bulliform phytoliths; **d** hair phytoliths. **a***B. glabra*; **b***C. aciculatus*; **c d***Z. mays*. **1**, end view; **2**, lateral view. **a1**, turned slightly to show the lateral side. Bar 10 μm, all pictures are in the same scale. **d** Bulliform phytoliths of subfamily Panicoideae, morphotypes observed in only one species and those with irregular morphology. **a***T. latifolia*; **b***S. spontaneum*; **c***R. repens*; **d***I. aristatum*; **e***C. tortilis*; **f***E. ophiuroides*; **g***S. sinense*; **h***S. bicolor*. **1**, end view; **2**, lateral view. **a1**, a silicified neighboring bulliform cell attached. Bar 10 μm, all pictures are in the same scale

**Fig. 10:**
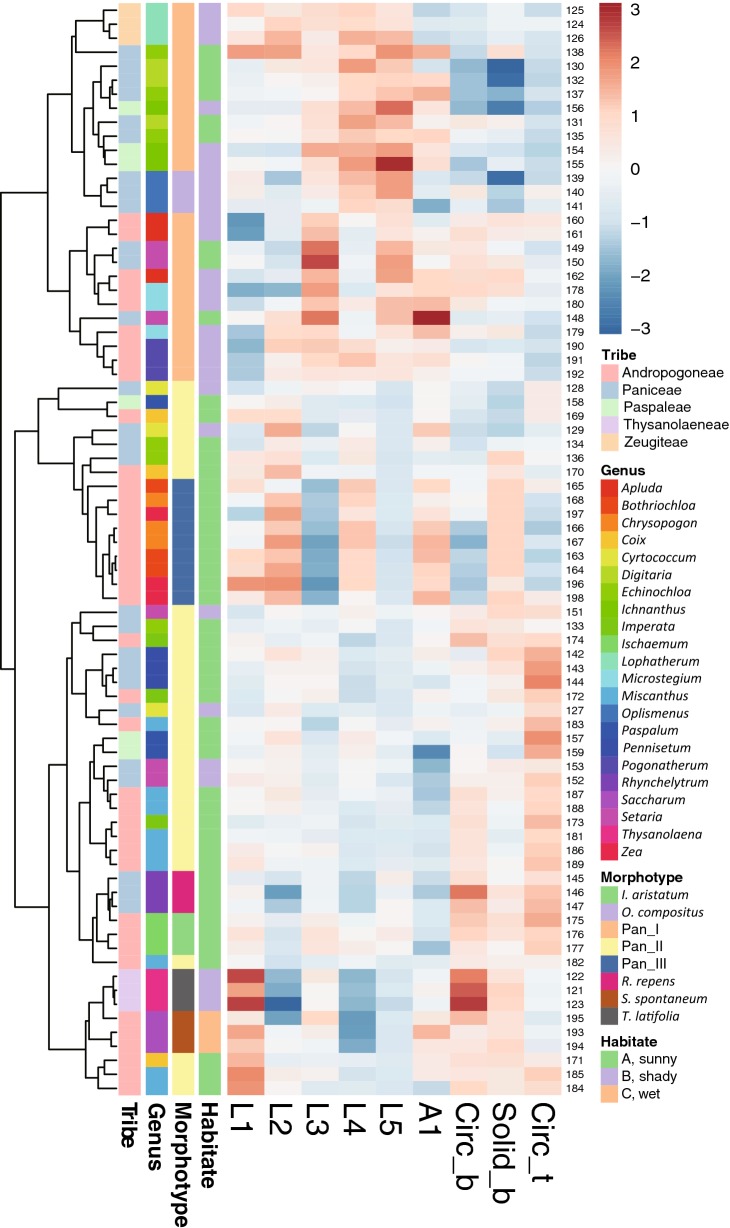
Cluster diagram and heat map of shape parameters from the analysis including measured samples of subfamily Panicoideae. The numbers are identical to the sample numbers in Table 1

Bulliform phytoliths of Panicoideae are characterized by a weakly cuneiform end profile: relatively long neighboring borders, wider tops and weakly curved bottoms (L2 and L4 large, Circ_b small; Fig. 3). Two major morphotypes, Pan_I and Pan_II, are prevalent in this subfamily. They are visually distinct from each other (Fig. 9) and are well separated by cluster analysis (Fig. 10). Pan_I type of bulliform phytoliths has a near rectangular end profile (Fig. 9A, end view). The grain is thick (L5 large) and therefore usually positioned on the lateral side (Fig. 9A, lateral view), which is rectangular with the bottom straight or slightly curved. The surface of the bottom carries obvious scale-like indentation marks which can be observed from the end and lateral sides (Fig. 9A). When viewed from the top (Fig. 9A, top view) and properly focused, the grain is elongate polygonal, and its bottom indentations appears reticulate.

The end profile of Pan_II bulliform phytoliths is rectangular to weakly cuneiform, usually slightly widened toward the bottom and asymmetrical (Fig. 9B). The bottom is curved and has irregular indentations. The lateral side (Fig. 9B, lateral view) shows that the thickness of the bulliform phytolith is usually smaller than the length (L5 small, Fig. 10). Most species of Panicoideae carry predominantly one of the two bulliform phytolith types, with occasional presence of the other type (1 to 5% of total bulliform phytoliths in one plant). In some species both types exist in equal amounts in the same plant (*E. colona* and *E. crus*-*galli*).

Typical Pan_II bulliform phytoliths have the width smaller than length (L3 < 1), and the width usually varies within the same plant, depending on the numbers of cells in one bulliform cell congregate. In some species, the majority of the bulliform phytoliths are equally narrow in width (*B. glabra*, *C. aciculatus*, and *Z. mays*; Fig. 9C). The thickness of the bulliform phytolith is similar to the width and the phytolith vaguely resembles a square pillar. In the cluster analysis this type of bulliform phytoliths formed a group (large L2, small L3, Fig. 10), and were designated type Pan_III. Large trichomes are frequently enclosed inside the bulliform cell congregates in this type of bulliform cell arrangement. In *Z. mays*, the hair associated with bulliform cells has a distinct shape and is silicified (Fig. 9C-d). It is tapered toward the top and slightly bent near the base.

Bulliform phytoliths of *T. latifolia* and *S. spontaneum* have morphology deviated from the typical Panicoideae. Bulliform phytoliths of *T. latifolia* are clearly similar to those of Arundinoideae, indicated by PCA and the clustering analysis (samples no. 121–123, Figs. 2 and 4). They are large, thin, with round bottoms and pointed tops (Fig. 9D-a). Bulliform phytoliths of *S. spontaneum* share these characters; however, they differ by having long and straight neighboring borders and irregularly undulate bottoms (Fig. 9D-b). When compared within Poaceae, bulliform phytoliths of *S. spontaneum* were similar to those of Bambusoideae (Figs. 2 and 4). In the analysis of Panicoideae, bulliform phytoliths of these two species were clustered in a group (Fig. 10).

Bulliform phytoliths of *R. repens* (Fig. 9D-c) and *I. aristatum* (Fig. 9D-d) also have prominent round bottoms (large Circ_b). Both are visually distinct because of the symmetrically curved bottoms and tops, and curved neighboring borders. The two differ by the width of the tops (L4): the tops of grains of *R. repens* are narrow, while those of *I. aristatum* are wide. They were grouped together in the cluster analysis (Fig. 10).

Among the Pan_I bulliform phytoliths, a special character was noticed in those of *O. compositus*. The mesophyll cells below the bulliform cells are large; therefore, there are prominent round and deep indentation marks on the bottoms of the bulliform phytoliths (Fig. 9A-j). This character could not be described well by shape parameters. However, it is visually distinct and present in all bulliform phytoliths of *O. compositus*. The same distinct character is present in silicified hair of *O. compositus* (Fig. 9A-k). The enlarged base of the funnel-shaped hair is deeply sharp serrate, similar to the indentation marks on the bottoms of the bulliform phytoliths. The hair is thin, and the top is narrow, flat, and usually broken. This distinct hair phytolith type is present in both accessions of *O. compositus* (Table 1), and is extremely abundant in the hairy variety (IC084). Another atypical feature of type Pan_I is present in the bulliform phytoliths of *P. crinitum* (Fig. 9A-i): round protrusions (papillae) on the top. Similar protrusions are present on the adaxial surface of long cells in the same plant. Not all the bulliform phytoliths from the same plant have round protrusions; nevertheless this character had not been observed in other species.

Bulliform phytoliths of *C. tortilis*, *E. ophiuroides*, *S. bicolor*, and *S. sinense* are irregular and variable in shape (Fig. 9D-e to h). Partial Pan_II character can be recognized in some grains (such as Fig. 9D-e); however, consistent features are lacking. Like those observed in Pooideae, this type of phytoliths can barely be recognized as bulliform cell in origin although the size matches.

The alpine dwarf form *M. sinensis* has bulliform phytoliths similar to those of the lowland counterpart (Fig. 9B-j, k). A pattern emerged when growing environments were mapped on to the dendrogram (Fig. 10). Most shady and partial shady species have type Pan_I bulliform phytoliths, whereas the species preferring exposed locations mostly have type Pan_II or Pan_III bulliform phytoliths.

#### Subfamily Micrairoideae

**Fig. 11:**
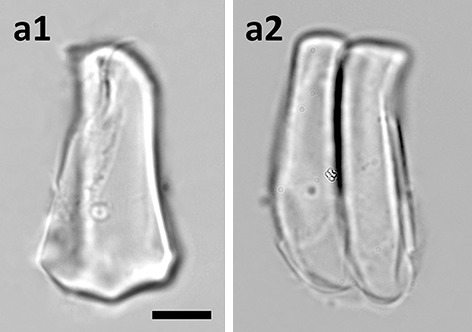
Bulliform phytoliths of subfamily Micrairoideae. **a***I. myositis*. **1**, end view; **2**, lateral view. **a2**, two grains attached. Bar 10 μ**m**, all pictures are in the same scale

The bulliform phytoliths of *I. myosotis* resemble Pan_III type visually (Fig. 11). The similarity was also indicated by PCA and cluster analysis (samples no. 199–201, Figs. 2 and 4).

#### Subfamily Arundinoideae

**Fig. 12:**
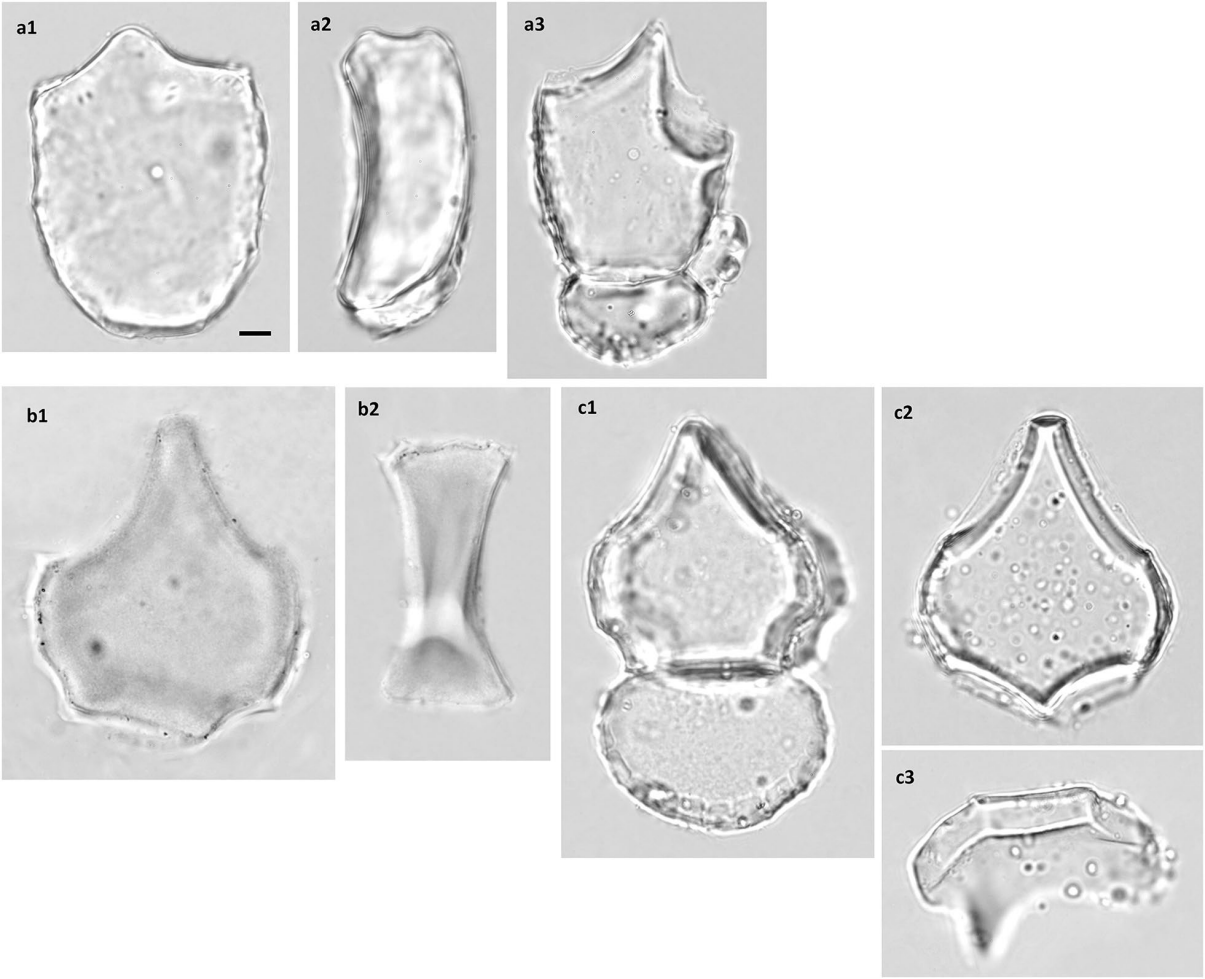
Bulliform phytoliths of subfamily Arundinoideae. **a***Phragmites* sp.; **b***A. donax*; **c***A. formosana*. **1**, **a3**, **c2**, end view; **2**, lateral view; **c3**, top view. **a3**, **c1**, silicified clear cells attached. Bar 10 μm, all pictures are in the same scale

The bulliform phytoliths of species of *Arundo* and *Phragmites* are characterized by being large, thin, and with pointed top (large L1, small L4, and small L5, Figs. 3, 12). Bulliform phytoliths of *Phragmites* sp. have round and deeply protruding bottoms, and relatively small neighboring bulliform phytoliths (Fig. 12-a). The grains sometimes have asymmetrical neighboring borders (Fig. 12-a3). Occasionally clear cells are silicified and remain attached to the bulliform cells (Fig. 12-a3). They do not leave indentation/curve marks on the bottoms of the bulliform phytoliths. Unlike those of *Phragmites* sp., the bottoms of bulliform phytoliths of *Arundo* sp. are bordered by 2-5 large-size clear cells, leaving clear large, curvy marks (Fig. 12-b, c). The clear cells of *Arundo* sp. are much larger than those of *Phragmites* sp., and are sometimes silicified (Fig. 12-c1). In addition, the neighboring bulliform phytoliths of *Arundo* sp. are proportionally larger comparing to those of *Phragmites* sp. (L2 larger).

*Phragmites* sp. and *A. formosana* from various habitats were sampled for bulliform phytoliths, and no morphological differences to distinguish habitats were observed. It should be noted that most of species in this subfamily were not sampled, and the description of the bulliform phytoliths above may not apply to the whole subfamily.

#### Subfamily Chloridoideae

**Fig. 13:**
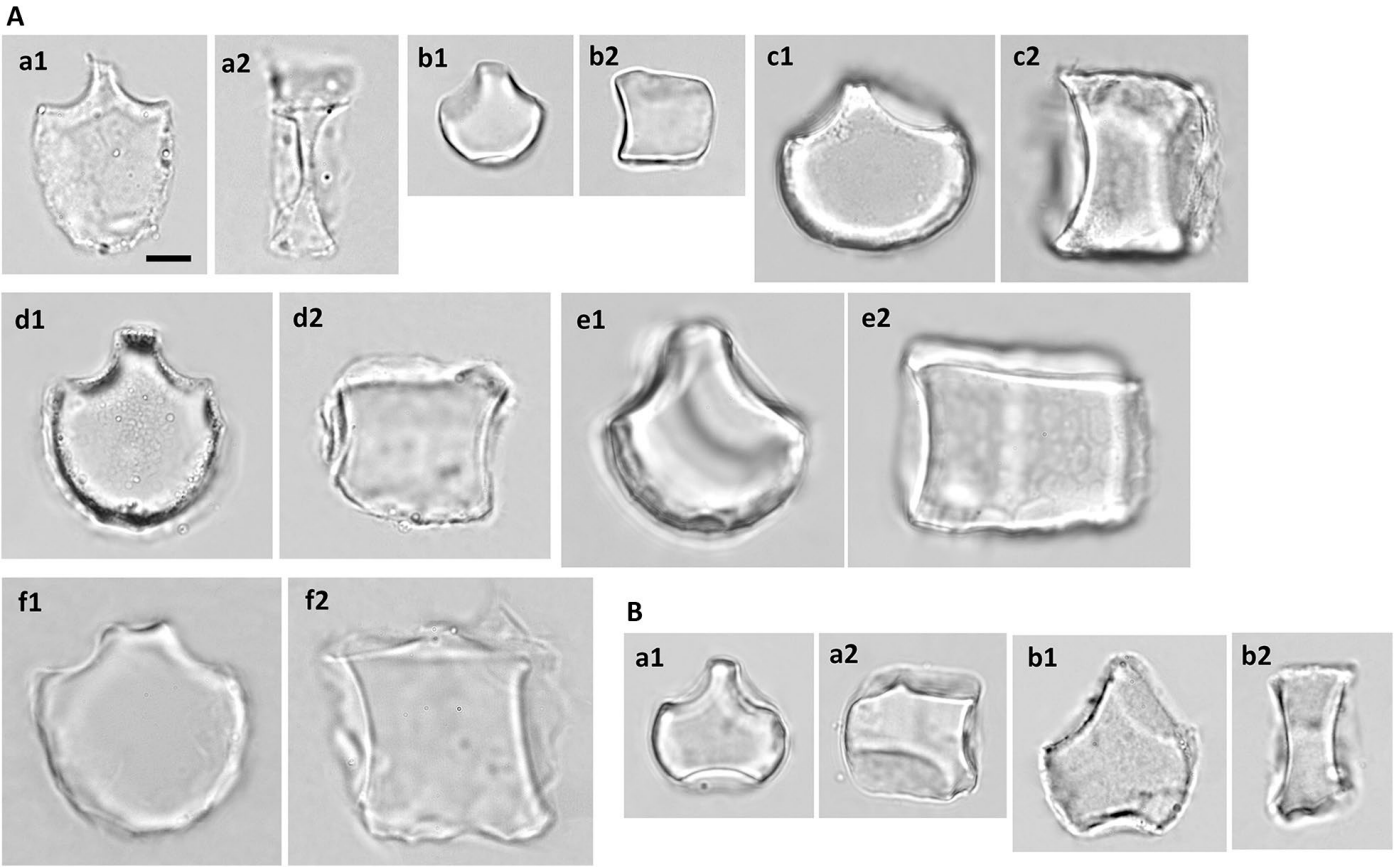
Bulliform phytoliths of subfamily Chloridoideae. A, morphotype Chl_I; B, morphotype Chl_II. **A a***E. amabilis*; **b***Eragrsotis* sp.; **c***S. indicus*; **d***C. barbata*; **e***D. aegyptium*; **f***E. indica*. **B a***Z. matrella*; **b***C. dactylon*. **1**, end view; **2**, lateral view. Bar 10 μm, all pictures are in the same scale

Bulliform phytoliths of Chloridoideae have an almost circular end profile. The two neighboring bulliform phytoliths are much smaller than the median one (L2 small, Fig. 3), leaving a pair of strong curve marks on the sides of a pointed top (Fig. 13). Thickness of the bulliform phytoliths usually varies greatly in the same plants. In this study, two morphotypes were recognized in this subfamily, Chl_I and Chl_II. These two morphotypes are similar except that Chl_II has one large indentation mark on the center of the bottom (Fig. 13B, end view). The concave indentation is the impression mark of a single large clear cell (Hsu et al. 2000; Watson et al. 1992). On the contrary, in the species possessing Chl_I bulliform phytolith type, several clear cells are adjacent to the median bulliform cell and do not cause large indentations on the bulliform phytoliths.

## Discussion

### Grass bulliform phytoliths are shaped up by the surrounding cells

Likely because of its large size, it is easy to discern various impression marks caused by the surrounding cells on a bulliform phytolith. This morphological characteristic is distinct from that of other cell types. Multiplicity is not common; extremely different bulliform phytolith morphotypes seldom occur in the same plant. Within a plant, there is usually one typical form, and variations are mostly from the small disparities in the nine shape parameters. The way the bulliform cells are arranged (forming a group or not, the number of cells in a group) and the size and shape of cells in contact decide the shape of a median bulliform phytolith, and these anatomical features are typically consistent within a subfamily.

In Oryzoideae and Bambusoideae, bulliform cells are usually arranged in groups of 3 or 5. Although not observed here, an electronic microscopic image of *Z. latifolia* bulliform phytoliths was shown in a previous study (Motomura et al. 2010). Sampled species of *Bambusa*, *Dendrocalamus*, and *Pleioblastus* have bulliform phytoliths similar to those of previously observed species of corresponding genera (Gu et al. 2016; Miyabuchi and Sugiyama 2016; Sase and Hosono 2001). The *Sasa* type defined in the previous studies (Miyabuchi and Sugiyama 2016) is asymmetrical, with one neighboring border straight and long, the other arched and short. These are the bulliform phytoliths from a group of four bulliform cells, the central two are large and the lateral two are extremely small (Motomura et al. 2004). Among our bamboo samples, this type of asymmetrical bulliform phytolith was observed mostly in *P. japonia* and *P. usawai* (Fig. 6A-h3). The amount may reach 10% of total bulliform phytoliths in one plant in maximum. It was occasionally observed in other bamboo samples, and the occurrence was not restricted in species with particular bulliform phytolith morphotypes. It is not clear whether this type of asymmetrical bulliform phytoliths are the dominant type in *Sasa* sp.

At least two major types of bulliform cell arrangement patterns exist in Panicoideae. Bulliform phytolith Pan_I resembles a long cell enlarged toward the mesophyll tissues. The leaf transverse sections of the grasses carrying this morphotype show that the bulliform cells gradually enlarge from veins to intercostal zones (leaf transverse section of *Lophatherum* in Watson et al. 1992), and except for those near veins, bulliform cells in the same intercostal zone differ not much in size. On the contrary, bulliform cells of grasses with Pan_II phytoliths are usually congregated as a group of 3 to several (transverse section of *Miscanthus* in Hsu et al. 2000). In some species of Panicoideae both Pan_I and Pan_II are present, suggesting that both types of bulliform cell arrangement patterns can occur in the same plant. Morphotype Pan_III is similar to Pan_II except for being consistently narrow in width. Leaf anatomy shows that the bulliform cells are arranged in a group of five or more, and all bulliform cells are about the same size, as the leaf transverse section of *Z. mays* shown in Hsu et al. (2000). Several non-typical forms occur in Panicoideae. At least for *S. spontaneum* hybridization could be the cause of forming bulliform phytoliths of unique shape since intergeneric hybrids were reported for *Sccharum* (Watson et al. 1992). Previously defined bulliform morphotypes—Paniceae type and *Miscanthus* type (Miyabuchi and Sugiyama 2016)—correspond to Pan_I and Pan_II types of this study.

The size and arrangement pattern of clear cells play a major role in shaping the bulliform phytoliths in Arundinoideae and Chloridoideae. In some species, the clear cells are enlarged to the size of several mesophyll cells. The clear cells arrangement patterns are consistent; the presence of both types of bulliform phytoliths—bottom with or without large concave indentations—in the same plant had not been observed. Clear cells are present in other subfamilies, but they do not leave large concave marks on bulliform phytoliths as observed in these two subfamilies. Bulliform phytoliths of *Phragmites* sp. and *Zoysia* sp. were recognized in several previous studies (Bowdery 1999; Inoue et al. 2016; Lu et al. 2006; Miyabuchi and Sugiyama 2016). Electron micrographs of bulliform phytoliths of *Hakonechloa macra* (Japan-endemic) and *Molinia japonica* have been shown in Motomura et al. (2010).

### Differential occurrence of bulliform phytoliths in grass subfamilies

In terms of silica deposition in bulliform cells, Bambusoideae is the most consistent. Abundant bulliform phytoliths were present in almost all sampled bamboos (Table 1). However, they were mostly collected from the same garden, so the factors from soil condition that influence bulliform phytolith production are not as various as those collected from the wild. Strikingly, hardly any bulliform phytolith was observed in Pooideae. Part of the reason is that some species do not form well developed bulliform cells (Hsu et al. 2000). Further investigation is needed to confirm whether in Pooideae, or in other temperate species, bulliform cells are in general not well developed or silicified. The process of bulliform cell formation/silicification in Pooideae is likely different from other subfamilies.

### Distinguishing similar Poaceae bulliform phytolith morphotypes

The rectangular morphotypes, Pan_I, Pan_II, and Pan_III, usually will not be confused with other round-bottomed (cuneiform) morphotypes. Among those with round bottoms, bulliform phytoliths of *Arundo* sp., *Phragmites* sp., and Chloridoideae are unique: their range of variation is limited, and they seldom resemble other morphotypes. Bulliform phytoliths of *T. latifolia* are large, thin, with narrow tops like those of *Arundo* sp. and *Phragmites* sp. Nevertheless the three can be easily differentiated by L2 and presence/absence of the large concave marks on the bottom. On the contrary, some of the type Bam_III grains (Fig. 6C) can be very similar to rice bulliform phytoliths. However, they can be distinguished by surface sculpture (parallel grooves vs. scale like indentations) on the bottoms.

Thick bulliform phytoliths are frequently oriented on the lateral side and cannot be properly identified without re-position. Thickness of bulliform phytoliths of Chloridoideae varies greatly. The thick ones usually reveal only the lateral side and appear parallelepipedal. Their end profiles will show the true identity. The pointed top will appear as one sharp parallel ridge on the top profiles, another distinct character to recognize bulliform phytoliths of Chloridoideae.

Morphotype Pan_I can be confused with the phytoliths of neighboring bulliform cells. However, the top profile of Pan_I is rectangular to polygonal, and the bottom has scale-like indentations. Phytoliths of neighboring bulliform cells do not have these characters.

### Distinguishing grass bulliform phytoliths from other blocky phytoliths

Conifers produce blocky polyhedral phytoliths similar to grass bulliform phytoliths, and it was reported that they can be distinguished from each other (An 2016). Members of Zingiberales produce tabular phytoliths with a sculptured base (Chen and Smith 2013). Plants of Miombo woodlands, at least those of Amaranthaceae, Annonaceae, Clusiaceae, Ebenaceae, and Fabaceae, may produce blocky, rectangular to polyhedral phytoliths (Mercader et al. 2009). Grass bulliform phytoliths Pan_I type is potentially not differentiable from the above mentioned blocky phytoliths. A vaguely cuneiform phytolith type was observed in Cyperaceae (Novello et al. 2012). It (Blo-5) resembles Pan_II type defined in this study judging from the image provided. Bulliform cells of Cyperaceae may arrange in a way similar to some Panicoid grasses (Martins and Alves 2009). It is possible that the bulliform phytoliths of Cyperaceae resemble Pan_II bulliform phytolith type. Phytoliths similar to the round bottom cuneiform types (large Circ_b) have not been reported from plants other than Poaceae. Little is known about the silicification of bulliform cells of other monocots.

### Hair phytoliths

Phytoliths of trichomes were present in most observed grass species (Table 1). Several common morphotypes of trichome phytoliths were observed in this study, among them were two that have not been previously described (Table 1; Fig. 9A-k, C-d). A brief survey on trichome phytoliths of some American grasses (Brown 1984) had shown that they are potentially taxonomically informative. A systematic description of grass trichome morphotypes may provide useful information for sedimentary study.

### Association of bulliform phytolith morphotypes with habit in Bambusoideae

Although seemingly variable and not easily delimited into types, morphology of bulliform phytoliths shows correlation to growth habit (size of stems) in the subfamily Bambusoideae. The most obvious pattern is that Bam_IV type is present only in tall, large bamboos. Bam_IV is easily recognizable, and the existence in the sediments could be a good indication of the presence of tall bamboos. Comparing soil phytolith assemblages from various native bamboo forests may further confirm this observation. Four major bamboo lineages were recognized by sequence data: paleotropical woody, neotropical woody, temperate woody, and herbaceous bamboos (Kelchner 2013). It would be of particular interest to observe the bulliform phytoliths of herbaceous bamboos, Tribe Olyreae, which had not been sampled in this study.

### Association of bulliform phytolith morphotypes with habitats

Chloridoideae produces easily recognizable bulliform phytoliths, and this subfamily is mostly short grasses inhabiting arid grasslands. Tall reed-like plants growing near water (*Arundo* sp., *Phragmites* sp., and *S. spontaneum*) all produce distinct bulliform phytolith types. These are examples of unequivocal association between bulliform phytolith types and habitats. Among the subfamilies with the round bottomed cuneiform bulliform phytoliths, a clear trend is identified: C3 grasses are associated with Oryzoideae, Bambusoideae, and Arundoideae types; C4 grasses are associated with Chloridoideae types (Table 1).

Our data showed that Pan_I type bulliform phytoliths were frequently found in shady species. Comparing top soil phytolith assemblages is necessary to confirm this association. Among the shady Panicoid grasses, the cosmopolitan forest understory species *O. compositus* have unique bulliform and hair phytoliths. These phytolith types potentially can be used to infer the presence of shady forest habitats. Most grasses in high altitude mountains (> 2000 m) do not produce bulliform phytoliths, except for *M. sinensis* and *Y. niitakayamensis*. Their bulliform phytoliths probably would be the major bulliform phytolith counts in the top soils from high altitude regions. Bulliform phytoliths were seldom observed in halophytic species, except for the *Phragmites* sp. from salt marshes (Table 1).

Our observation indicated that the same species from different habitats produce the same bulliform phytolith morphotype (Table 1). The results would have been more conclusive if more grains were measured. Yet we confirmed that at least there is no immediate visual differentiation on the morphology due to differences in habitats.

### Use of bulliform phytoliths in environmental reconstruction

Although distinct morphotypes can infer the presence of specific taxa, comparing soil/sediment phytolith assemblages remains to be the essential way to reconstruct paleoenvironments. Most present-day comparative works on modern soil phytolith assemblages recognized two types of bulliform phytoliths, cuneiform and parallepiped. Our study indicated that bulliform phytoliths may provide a lot more information when properly classified. The shape parameters set up in this study are useful tools for delimiting morphotypes, considering that the shape variations are continuous and immediate visual distinction is sometimes difficult. Surface texture of the bulliform phytoliths, and the presence of phytoliths of special cell types (e.g., arm cells and fusoids) are also valuable, if they have been preserved in the sediments.

In a study of modern soil phytolith assemblages in western Africa, fan-shaped index (Fs) (%), fan-shaped vs. sum of characteristic phytoliths, was found to be increasing toward the more arid regions (Bremond et al. 2005). However, the same trend was not observed from the soils in Chad, central Africa (Novello et al. 2012). How a cuneiform phytolith type was defined is likely the main cause of this inconsistency. Bulliform phytoliths of *Phragmites* sp. and Chloridoideae would have been both classified as perfect cuneiform although they occupy different ecological niches. One or two shape parameters from this study can be effectively used to differentiate the two types of cuneiform phytoliths. Refined morphotype classification can markedly improve the accuracy of past flora/climate reconstruction. Other climate indices such as the warm index (Zhang et al. 2019) that incorporate data on cuneiform/bulliform phytoliths could benefit from further re-examination.

The factors that influence the formation of bulliform phytoliths should be taken into consideration when bulliform phytoliths are used as tools for environmental reconstruction. Comparing to short cells, silica uptake/deposition in bulliform cells is more sensitive to biotic or abiotic conditions. Besides floral composition, bulliform phytolith ratio in the phytolith assemblage may have reflected the overall effects of silica availability in soils. Knowledge of solid silica formation in plant cells is important for making accurate interpretation on soil phytolith assemblage data.

## Conclusions

In this study, a systematic description of the morphology of Poaceae bulliform phytoliths was accomplished. Presence or absence of bulliform phytoliths was recorded, and morphotypes were defined via morphometric methods. The morphology of bulliform phytoliths is in general consistent within subfamilies, suggesting a common evolutionary origin. Usually the end view of the grains contains recognizable features: Oryzoideae and Bambusoideae have a semi-circular to fan-shaped end profile; Panicoideae and Micrairoideae have a rectangular to weakly cuneiform end profile; Arundinoideae and Chloridoideae have an oval to circular end profile. Bulliform phytoliths were not observed in most species of Pooideae. There are typically two to four common bulliform phytolith types within a subfamily; species-level recognition is rare. Besides bulliform phytoliths, two unusual trichome phytolith types associated with bulliform cells were identified. Silicified arm cells and fusoids of Bambusoideae were reported for the first time.

When morphotype classification is properly refined, association between types and habit/habitats can be more readily revealed. Bulliform phytolith types may differentiate growth habit of bamboos. Large, thin, and pointed bulliform phytoliths are common in reed-like grasses grown near water; Chloridoideae types of bulliform phytoliths are good C4 plant indicators. Additional advantage of morphometric measurement is that further observation of bulliform phytoliths can be compared by the same shape parameters, and adjustments on morphotype classification can be easily made. The Poaceae bulliform phytoliths had never been fully investigated in Taiwan; therefore, the results of this study are an essential addition to local paleoenvironmental research. This study provides important references for interpreting the phytolith assemblage data, especially when large size phytoliths are dominant in the sediments.

## Supplementary information

**Additional file 1: Appendix S1.** Morphometric measurement of Poaceae bulliform phytoliths.

## Data Availability

All data generated or analysed during this study are included in this published article.

## References

[CR71] An X (2016). Morphological characteristics of phytoliths from representative conifers in China. Palaeoworld.

[CR1] An X, Lu H, Chu G (2015). Surface soil phytoliths as vegetation and altitude indicators: a study from the southern Himalaya. Sci Rep.

[CR2] Barboni D, Bremond L (2009). Phytoliths of East African grasses: an assessment of their environmental and taxonomic significance based on floristic data. Rev Palaeobot Palynol.

[CR3] Bowdery D (1999). Phytoliths from tropical sediments: reports from Southeast Asia and Papua New Guinea. Bull Indo-Pac Prehist Assoc.

[CR4] Bremond L, Alexandre A, Peyron O, Guiot J (2005). Grass water stress estimated from phytoliths in West Africa. J Biogeogr.

[CR5] Brown DA (1984). Prospects and limits of a phytolith key for grasses in the central United States. J Archaeol Sci.

[CR6] Cabanes D, Shahack-Gross R (2015). Understanding fossil phytolith preservation: the role of partial dissolution in paleoecology and archaeology. PLoS ONE.

[CR7] Chen YP (2009). Phytolith analysis on Taiwan’s living plants and prehistoirc potteries (台灣現生植物與史前陶片的矽酸體分析). J Natl Taiwan Museum.

[CR8] Chen ST, Smith SY (2013). Phytolith variability in Zingiberales: a tool for the reconstruction of past tropical vegetation. Palaeogeogr Palaeoclimatol Palaeoecol.

[CR9] Dey SB, Ghosh R, Shekhar M, Mukherjee B, Bera S (2015). What drives elevational pattern of phytolith diversity in *Thysanolaena maxima* (Roxb.) O. Ktze? A study from the Darjeeling Himalayas. Flora.

[CR10] Ellis RP (1976). A procedure for standardizing comparative leaf anatomy in the Poaceae. I. The leaf-blade as viewed in transverse section. Bothalia.

[CR11] Ellis RP (1979). A procedure for standardizing comparative leaf anatomy in the Poaceae. II. The epidermis as seen in surface view. Bothalia.

[CR12] Feng Y, Jie D, Me Guo, Dong S, Chen X, Liu H, Liu L, Li N (2017). Phytolith loss and enrichment in soil phytolith assemblages revealed by comparisons of phytoliths in vegetation and surface soils of altitudinal belts in the Changbai Mountains, Northeast China. Flora.

[CR13] Fujiwara H, Pearsall DM, Piperno DR (1993). Research into the history of rice cultivation using plant opal analysis. Current research in phytolith analysis, application in archaeology and paleoecology.

[CR14] Gu Y, Zhao Z, Pearsall DM (2013). Phytolith morphology research on wild and domesticated rice species in East Asia. Quat Int.

[CR15] Gu Y, Liu H, Wang H, Li R, Yu J (2016). Phytoliths as a method of identification for three genera of woody bamboos (Bambusoideae) in tropical southwest China. J Archaeol Sci.

[CR16] Hart TC (2016). Issues and directions in phytolith analysis. J Archaeol Sci.

[CR17] Honaine MF, Osterrieth ML (2012). Silicification of the adaxial epidermis of leaves of a panicoid grass in relation to leaf position and section and environmental conditions. Plant Biol.

[CR18] Hsieh CF, Shen CF (1994) Introduction to the flora of Taiwan, 1: geography, geology, climate, and soils. Flora of Taiwan, vol 1, second edn. Editorial Committee, Department of Botany, National Taiwan University, Taipei, Taiwan. http://tai2.ntu.edu.tw/ebook.php?ebook=Fl.%20Taiwan%202nd. Accessed 18 Dec 2019

[CR19] Hsu CC, Kuoh CS, Liu HY (2000) Gramineae (Poaceae). Flora of Taiwan, vol. 5, 2nd edn. Editorial Committee, Department of Botany, National Taiwan University, Taipei, Taiwan. http://www.efloras.org/flora_page.aspx?flora_id=1050. Accessed 18 Dec 2019

[CR20] Huan X, Lu H, Wang C, Tang X, Zuo X, Ge Y, He K (2015). Bulliform phytolith research in wild and domesticated rice paddy soil in South China. PLoS ONE.

[CR21] Inoue J, Okunaka R, Kawano T (2016). The relationship between past vegetation type and fire frequency in western Japan inferred from phytolith and charcoal records in cumulative soils. Quat Int.

[CR22] Neumann K, Strömberg CA, Ball T, Albert RM, Vrydaghs L, Cummings LS, International Committee for Phytolith Taxonomy (2019). International code for phytolith nomenclature (ICPN) 2.0. Ann Bot.

[CR23] Issaharou-Matchi I, Barboni D, Meunier JD, Saadou M, Dussouillez P, Contoux C, Zirihi-Guede N (2016). Intraspecific biogenic silica variations in the grass species *Pennisetum pedicellatum* along an evapotranspiration gradient in South Niger. Flora.

[CR24] Jenkins E (2009). Phytolith taphonomy: a comparison of dry ashing and acid extraction on the breakdown of conjoined phytoliths formed in *Triticum durum*. J Archaeol Sci.

[CR25] Kaufman PB, Dayanandan P, Franklin CI, Takeoka Y (1985). Structure and function of silica bodies in the epidermal system of grass shoots. Ann Bot.

[CR26] Kelchner SA (2013). Higher level phylogenetic relationships within the bamboos (Poaceae: Bambusoideae) based on five plastid markers. Mol Phylogen Evol.

[CR27] Kumar S, Soukup M, Elbaum R (2017). Silicification in grasses: variation between different cell types. Front Plant Sci.

[CR28] Li R, Fan J, Carter J, Jiang N, Gu Y (2017). Monthly variations of phytoliths in the leaves of the bamboo *Dendrocalamus ronganensis* (Poaceae: Bambusoideae). Rev Palaeobot Palynol.

[CR29] Li WQ, Zhang MJ, Gan PF, Qiao L, Yang SQ, Miao H, Wang GF, Zhang MM, Liu WT, Li HF, Shi CH, Chen KM (2017). CLD1/SRL1 modulates leaf rolling by affecting cell wall formation, epidermis integrity and water homeostasis in rice. Plant J.

[CR30] Liu L, Jie D, Liu H, Gao G, Gao Z, Li D, Li N, Guo J, Qiao Z (2016). Assessing the importance of environmental factors to phytoliths of *Phragmites communis* in north-eastern China. Ecol Indicators.

[CR31] Lu H, Liu KB (2003). Morphological variations of lobate phytoliths from grasses in China and the south-eastern United States. Divers Distrib.

[CR32] Lu H, Liu Z, Wu N, BernÉ S, Saito Y, Liu B, Wang L (2002). Rice domestication and climatic change: phytolith evidence from East China. Boreas.

[CR33] Lu HY, Wu NQ, Yang XD, Jiang H, Liu KB, Liu TS (2006). Phytoliths as quantitative indicators for the reconstruction of past environmental conditions in China I: phytolith-based transfer functions. Quat Sci Rev.

[CR34] Ma JF, Yamaji N (2006). Silicon uptake and accumulation in higher plants. Trends Plant Sci.

[CR35] Martins S, Alves M (2009). Anatomical features of species of Cyperaceae from northeastern Brazil. Brittonia.

[CR36] Mercader J, Bennett T, Esselmont C, Simpson S, Walde D (2009). Phytoliths in woody plants from the Miombo woodlands of Mozambique. Ann Bot.

[CR37] Metcalfe CR (1960). Anatomy of the monocotyledons. 1. Gramineae.

[CR38] Metsalu T, Vilo J (2015). ClustVis: a web tool for visualizing clustering of multivariate data using Principal Component Analysis and heatmap. Nucleic Acids Res.

[CR39] Miyabuchi Y, Sugiyama S (2016). 90,000-year phytolith records from caldera rim to western foot of Aso Volcano, Japan: implications for vegetation history since catastrophic eruption. Quat Int.

[CR40] Motomura H, Fujii T, Suzuki M (2004). Silica deposition in relation to ageing of leaf tissues in *Sasa veitchii* (Carrière) Rehder (Poaceae: Bambusoideae). Ann Bot.

[CR41] Motomura H, Yonekura K, Kondo E (2010). Diversity and descriptive terminology of morphological features in bulliform cell phytoliths of grasses and bamboos (イネ科植物の泡状細胞珪酸体形状の多様性と記載用語の提案). Jap J Hist Bot.

[CR42] Neumann K, Fahmy AG, Müller-Scheeßel N, Schmidt M (2015). Taxonomic, ecological and palaeoecological significance of leaf phytoliths in West African grasses. Quat Int.

[CR43] Novello A, Barboni D, Berti-Equille L, Mazur J-C, Poilecot P, Vignaud P (2012). Phytolith signal of aquatic plants and soils in Chad, Central Africa. Rev Palaeobot Palynol.

[CR44] Osborne CP, Salomaa A, Kluyver TA, Visser V, Kellogg EA, Morrone O, Vorontsova MS, Clayton WD, Simpson DA (2014). A global database of C4 photosynthesis in grasses. New Phytol.

[CR45] Parry DW, Smithson F (1958). Silicification of bulliform cells in grasses. Nature.

[CR46] Pearsall DM (2000) Chapter 5—phytolith analysis. In: Paleoethnobotany, a handbook of procedures, 2nd edn. Academic Press, San Diego, pp 311–438. 10.1016/b978-0-12-548040-6.50009-5

[CR47] Pearsall DM, Piperno DR, Dinan EH, Umlauf M, Zhao Z, Benfer RA (1995). Distinguishing rice (*Oryza sativa* Poaceae) from wild *Oryza* species through phytolith analysis: results of preliminary research. Econ Bot.

[CR48] Piperno DR (2006). Phytoliths: a comprehensive guide for archaeologists and paleoecologists.

[CR49] Rasband WS (1997–2016) ImageJ, U. S. National Institutes of Health, Bethesda. https://imagej.nih.gov/ij/. Accessed 18 Dec 2019

[CR50] Renvoize SA (1982). A survey of leaf-blade anatomy in grasses II. Arundinelleae. Kew Bull.

[CR51] Renvoize SA (1982). A survey of leaf-blade anatomy in grasses III. Garnotieae. Kew Bull.

[CR52] Renvoize SA (1982). A survey of leaf-blade anatomy in grasses. I. Andropogoneae. Kew Bull.

[CR53] Renvoize SA (1983). A survey of leaf-blade anatomy in grasses IV. Eragrostideae. Kew Bull.

[CR54] Renvoize SA (1985). A survey of leaf-blade anatomy in grasses V. The Bamboo Allies. Kew Bull.

[CR55] Renvoize SA (1985). A survey of leaf-blade anatomy in grasses VI Stipeae. Kew Bull.

[CR56] Renvoize SA (1985). A survey of leaf-blade anatomy in grasses. VII Pommereulleae, Orcuttieae & Pappophoreae. Kew Bull.

[CR57] Renvoize SA (1986). A survey of leaf-blade anatomy in grasses IX. Centothecoideae. Kew Bull.

[CR58] Renvoize SA (1986). A survey of leaf-blade anatomy in grasses VIII. Arundinoideae. Kew Bull.

[CR59] Renvoize SA (1987). A survey of leaf-blade anatomy in grasses X: Bambuseae. Kew Bull.

[CR60] Sangster AG, Parry DW (1969). Some factors in relation to bulliform cell silicification in the grass leaf. Ann Bot.

[CR61] Sase T, Hosono M, Meunier JD, Colin F (2001). Phytolith record in soils interstratified with late quaternary tephras overlying the eastern region of Towada volcano, Japan. Phytoliths : applications in earth sciences and human history.

[CR62] Soreng RJ, Peterson PM, Romaschenko K, Davidse G, Zuloaga FO, Judziewicz EJ, Filgueiras TS, Davis JI, Morrone O (2015). A worldwide phylogenetic classification of the Poaceae (Gramineae). J Syst Evol.

[CR63] Spitzer M, Wildenhain J, Rappsilber J, Tyers M (2014). BoxPlotR: a web tool for generation of box plots. Nat Methods.

[CR64] Strömberg CAE (2005). Decoupled taxonomic radiation and ecological expansion of open-habitat grasses in the Cenozoic of North America. Proc Natl Acad Sci USA.

[CR65] Wang C, Udatsu T, Fuiiwara H (1996). Relationship between the shape of silica body from motor cells and morphological and physiological characters of grain for the discriminations of *indica* and *japonica* Rice in China. Jpn J Breed.

[CR66] Watson L, Macfarlane TD, Dallwitz MJ (1992) The grass genera of the world: descriptions, illustrations, identification, and information retrieval; including synonyms, morphology, anatomy, physiology, phytochemistry, cytology, classification, pathogens, world and local distribution, and references. Version: 11th December 2017. http://www.delta-intkey.com. Accessed 18 Dec 2019

[CR67] Wu MCY (1962). The classification of Bambuseae based on leaf anatomy. Bot Bull Acad Sinica.

[CR68] Wu Z, Raven PH, Hong D (1994) Flora of China. Science Press, Beijing; Missouri Botanical Garden, St. Louis. http://www.eflora.org. Accessed 18 Dec 2019

[CR69] Zhang XR, Du Y, Ma CM, Ping SF, Feng C, Cui AN (2019). Climatic controls on peat swamp formation and evolution since 1300 year BP as recorded by phytoliths in the Xishan Mountains, Jiangxi Province, China. Palaeogeogr Palaeoclimatol Palaeoecol.

[CR70] Zheng WJ, Zheng XP, Zhang CL (2000). A survey of photosynthetic carbon metabolism in 4 ecotypes of Phragmites australis in northwest China: Leaf anatomy, ultrastructure, and activities of ribulose 1,5-bisphosphate carboxylase, phosphoenolpyruvate carboxylase and glycollate oxidase. Physiol Plant.

