# Supplementary material for: Silicified bulliform cells of Poaceae: morphological characteristics that distinguish subfamilies
Source: Bot Stud. 2020 Mar 2;61:5. doi: 10.1186/s40529-020-0282-x (PMC7052108; doi:10.1186/s40529-020-0282-x)
Supplement: Supplementary file 1 — Additional file 1: Appendix S1. Morphometric measurement of Poaceae bulliform phytoliths. [file 40529_2020_282_MOESM1_ESM.pdf]

Appendix I. Morphometric measurement of Poaceae bulliform phytoliths.

Sample no.	Accession	L1	L2	L3	L4	L5	A1	Circ_b	Solid_b	Circ_t
1	IC054	41.53	0.49073	0.97255	0.24462	0.49771	140.9	0.74	0.99	0.1
2	IC054	53.75	0.49488	0.89098	0.21925	0.84688	144.16	0.82	0.99	0.07
3	IC054	53.98	0.50574	0.8379	0.25426	0.38811	138.63	0.77	0.97	0.07
4	IC226	47.9	0.69123	0.7762	0.31038	0.40251	166.52	0.638	0.994	0.2
5	IC226	51.82	0.69548	0.84658	0.32505	0.28966	154.89	0.623	0.992	0.173
6	IC226	47.03	0.74378	0.95684	0.25511	0.56857	179.53	0.614	1	0.226
7	IC223	49.22	0.67168	0.87871	0.34497	0.86408	168.5	0.653	1	0.102
8	IC223	56.63	0.6765	0.80099	0.33311	0.54441	173.23	0.573	1	0.185
9	IC223	48.67	0.64845	0.83008	0.39332	0.44422	147.72	0.68	0.991	0.123
10	IC221	59.45	0.64272	0.79983	0.22608	0.35324	171.07	0.685	0.97	0.199
11	IC221	55.2	0.69094	0.7413	0.35533	0.4433	179.31	0.583	0.994	0.306
12	IC221	60.12	0.67914	0.85978	0.28652	0.34065	178.52	0.63	0.998	0.271
13	IC222	59.38	0.63355	0.86359	0.26151	0.60997	180.1	0.708	1	0.092
14	IC222	53.91	0.61324	0.77462	0.28784	0.59785	181.23	0.68	1	0.269
15	IC222	60.17	0.71713	0.78677	0.26236	0.58734	185.54	0.702	1	0.166
16	IC218	37.14	0.57647	0.89634	0.21658	0.49919	155.62	0.762	0.997	0.107
17	IC218	40.93	0.62649	1.01513	0.19301	0.48388	158.25	0.66	0.991	0.157
18	IC218	40.78	0.63315	0.91687	0.24686	0.82075	165.62	0.644	1	0.02
19	IC199	42.56	0.61889	0.76057	0.2595	0.4758	171.38	0.611	0.985	0.147
20	IC199	42.01	0.54582	0.8548	0.12977	0.51583	177.8	0.685	0.987	0.112
21	IC199	37.13	0.568	0.80097	0.231	0.68301	176.01	0.691	1.001	0.139
22	IC198	49.99	0.58092	0.93939	0.13352	0.57752	162.05	0.724	0.996	0.166
23	IC198	47.85	0.57367	0.86667	0.26163	0.67732	172.46	0.665	0.989	0.179
24	IC198	53.38	0.54683	0.84882	0.18384	0.38048	156.12	0.748	0.998	0.087
25	IC200	40.96	0.45898	0.92749	0.24664	0.92236	169.68	0.775	0.995	0.075
26	IC200	42.84	0.48599	0.91573	0.19577	0.48273	165.72	0.741	1	0.026
27	IC200	45.77	0.58816	0.84619	0.1828	0.58444	162.72	0.694	0.993	0.345
28	IC225	47.79	0.49425	0.84934	0.26238	0.83051	162.56	0.789	0.989	0.111
29	IC225	47.32	0.55811	0.84954	0.22836	0.58812	170.82	0.777	1	0.156
30	IC225	54.21	0.47371	0.7447	0.31707	0.57904	164.49	0.789	0.999	0.037
31	IC203	46.91	0.55255	0.70795	0.22222	0.38393	158.86	0.758	0.991	0.097
32	IC203	50.29	0.517	0.7928	0.19814	0.40744	162.48	0.754	0.988	0.089
33	IC203	48.77	0.62579	0.71396	0.24411	0.64548	157.39	0.756	0.996	0.151
34	IC236	59.43	0.64378	0.83359	0.25071	0.66397	184.2	0.699	0.994	0.275
35	IC236	50.87	0.70474	0.89601	0.23738	0.40436	172.58	0.579	1	0.23
36	IC236	55.45	0.6321	0.87286	0.26508	0.54103	172.01	0.685	0.992	0.138
37	IC216	49.69	0.64359	0.87503	0.39489	0.58503	169.59	0.657	0.999	0.05
38	IC216	50.96	0.67406	0.94447	0.31955	0.68838	169.14	0.591	1	0.118
39	IC216	52.3	0.69426	0.71377	0.4262	0.5566	177.07	0.657	1	0.075
40	IC087	51.94	0.60377	0.79149	0.26903	0.23681	179.06	0.672	0.977	0.048
41	IC087	59.38	0.61671	0.88127	0.36556	0.28595	176.6	0.629	0.995	0.021
42	IC087	54.73	0.61538	0.81071	0.39306	0.27882	168.26	0.685	0.974	0.022
43	IC213	60.71	0.69363	0.79196	0.21194	0.30539	179.03	0.647	1	0.13
44	IC213	53.94	0.70245	0.80942	0.16537	0.36652	175.99	0.646	1	0.248
45	IC213	50.48	0.63689	0.93958	0.20789	0.39739	179.16	0.619	0.997	0.168
46	IC219	57.76	0.50121	0.92746	0.18704	0.50398	167.23	0.736	0.97	0.045
47	IC219	50.33	0.52533	0.87463	0.28646	0.6058	165.27	0.752	1	0.176
48	IC219	42.25	0.55811	0.99527	0.21998	0.71621	152.73	0.627	1	0.171
49	IC224	58.58	0.61932	0.77177	0.29131	0.44589	160.43	0.77	0.994	0.267
50	IC224	68.11	0.61547	0.79284	0.29333	0.28557	174.92	0.758	0.984	0.045
51	IC224	59.25	0.56827	0.7811	0.25648	0.42481	171.87	0.76	0.993	0.183
52	IC243	48.7	0.69302	0.83368	0.27241	0.54292	175.21	0.645	1	0.291
53	IC243	58.08	0.58833	0.84694	0.28237	0.60279	178.12	0.746	0.993	0.132
54	IC243	57.97	0.67173	0.76143	0.29384	0.41418	160.38	0.582	0.994	0.148
55	IC197	30.97	0.57346	0.98708	0.27216	0.97029	155.94	0.651	0.958	0.225
56	IC197	39.29	0.5775	0.84144	0.28554	0.61517	161.48	0.591	0.964	0.268
57	IC197	32.6	0.66564	0.90337	0.38778	0.99233	163.74	0.629	0.982	0.19
58	IC111	35.78	0.53829	0.81666	0.16632	0.39324	147.91	0.58	0.91	0.13
59	IC111	38.81	0.5715	0.98274	0.22994	0.32079	138.24	0.58	0.93	0.3

60	IC111	38.17	0.55122	0.90464	0.3481	0.58135	142.17	0.64	0.95	0.41
61	IC220	36.33	0.59125	0.93889	0.27206	0.52381	153.48	0.611	0.958	0.333
62	IC220	37.06	0.61017	1.0416	0.3462	0.47133	161.9	0.642	0.973	0.223
63	IC220	38.63	0.5897	0.94926	0.28797	0.40538	161.32	0.607	0.933	0.274
64	IC231	45.41	0.67474	0.83924	0.20152	0.43338	174.33	0.67	0.993	0.072
65	IC231	50.91	0.61756	0.70654	0.1315	0.41819	177.22	0.73	0.984	0.175
66	IC231	46.32	0.56455	0.92962	0.13307	0.52569	162.98	0.707	0.975	0.166
67	IC230	57.89	0.62239	0.65279	0.17756	0.4702	172.41	0.773	0.978	0.25
68	IC230	56.65	0.60388	0.68685	0.10871	0.71598	174.04	0.812	0.989	0.317
69	IC230	47.89	0.62017	0.72499	0.16187	0.54082	181.2	0.74	0.984	0.109
70	IC202	61.46	0.50163	0.67833	0.14248	0.24829	163.16	0.679	0.967	0.167
71	IC202	53.77	0.4934	0.7958	0.2136	0.28696	158.84	0.747	0.984	0.028
72	IC202	55.27	0.5768	0.78288	0.20153	0.31355	160.53	0.703	0.97	0.05
73	IC227	41.01	0.51865	0.79688	0.21512	0.44891	163.16	0.704	0.975	0.168
74	IC227	49.51	0.62129	0.68653	0.17182	0.46233	165.24	0.695	0.986	0.117
75	IC227	45.5	0.63253	0.75385	0.13294	0.74264	163.95	0.74	1	0.21
76	IC003	48.34	0.55978	0.69673	0.22298	0.51158	173.12	0.72	0.98	0.25
77	IC003	59.27	0.49232	0.71942	0.28659	0.36781	164.49	0.79	0.99	0.24
78	IC003	65.85	0.50554	0.61974	0.20779	0.29476	161.1	0.76	0.95	0.14
79	IC228	65.16	0.498	0.56446	0.12996	0.4314	154.23	0.696	0.901	0.261
80	IC228	55.12	0.58436	0.67852	0.1369	0.35232	147.19	0.602	0.877	0.336
81	IC228	67.61	0.52137	0.5724	0.10207	0.29685	165.76	0.673	0.902	0.218
82	IC232	67.59	0.50052	0.62509	0.13538	0.23909	150.88	0.667	0.925	0.133
83	IC232	59.98	0.55735	0.55919	0.15832	0.31344	163.39	0.734	0.971	0.262
84	IC232	71.46	0.43535	0.60188	0.1595	0.28505	150.23	0.699	0.932	0.243
85	IC212	92.65	0.52617	0.53114	0.11299	0.36373	168.67	0.749	0.973	0.369
86	IC212	72.08	0.59975	0.68979	0.08689	0.42411	179.25	0.697	0.922	0.479
87	IC212	80.97	0.59479	0.45992	0.10365	0.23009	177.41	0.672	0.904	0.319
88	IC215	83.38	0.52039	0.62173	0.09105	0.4302	156.72	0.727	0.919	0.385
89	IC215	73.79	0.52053	0.59629	0.09886	0.31956	163.44	0.787	0.966	0.302
90	IC215	82.4	0.51529	0.54636	0.06975	0.25704	157.64	0.647	0.878	0.488
91	IC250	60.82	0.45676	0.72476	0.13521	0.49194	178.69	0.642	0.861	0.05
92	IC250	61.68	0.60879	0.62305	0.08249	0.56372	168.08	0.743	0.97	0.287
93	IC250	69.32	0.50058	0.66287	0.08422	0.33569	176.35	0.782	0.973	0.306
94	IC245	68.35	0.55933	0.68237	0.10978	0.32612	154.68	0.605	0.882	0.447
95	IC245	61.72	0.58036	0.70334	0.08201	0.43082	155.39	0.708	0.96	0.345
96	IC245	70.51	0.56049	0.80343	0.05984	0.39569	168.2	0.673	0.945	0.349
97	IC211	61.52	0.55348	0.57412	0.10504	0.28771	159.08	0.743	0.966	0.394
98	IC211	64.22	0.5805	0.5844	0.2033	0.29773	173.68	0.688	0.94	0.103
99	IC211	58.88	0.59715	0.6053	0.21072	0.5197	170.7	0.77	0.992	0.392
100	IC239	60.72	0.75478	0.84552	0.26899	0.33926	177.56	0.555	0.96	0.245
101	IC239	57.64	0.71253	0.85947	0.31449	0.58917	179.81	0.626	0.976	0.165
102	IC239	72.3	0.77524	0.70705	0.27719	0.51107	166.94	0.615	0.992	0.108
103	IC246	49.93	0.47086	0.69818	0.17814	0.34849	165.61	0.732	0.959	0.178
104	IC246	43.74	0.5144	0.81802	0.11543	0.45107	172.1	0.754	0.991	0.287
105	IC246	45.95	0.52709	0.68792	0.15122	0.41436	162.79	0.766	0.99	0.013
106	IC214	71.92	0.49903	0.78281	0.15364	0.28991	147.08	0.711	0.932	0.024
107	IC214	62.98	0.65052	0.65306	0.12327	0.37949	151.61	0.735	0.969	0.072
108	IC214	68.64	0.56119	0.75554	0.06248	0.46766	155.45	0.786	0.984	0.048
109	IC233	59.11	0.61546	0.87616	0.25352	0.2585	175.45	0.635	0.984	0.209
110	IC233	50.47	0.57717	0.89776	0.22445	0.40638	165.13	0.713	0.968	0.374
111	IC233	69.53	0.68877	0.77247	0.22957	0.39839	172.05	0.653	1	0.195
112	IC251	36.44	0.46076	0.74177	0.14724	0.78979	175.98	0.787	1	0.013
113	IC251	46.38	0.52523	0.6796	0.20114	0.48491	167.18	0.77	0.99	0.062
114	IC251	48.33	0.43906	0.75295	0.19071	0.56818	169.82	0.808	0.981	0.054
115	IC156	38.04	0.59569	0.61462	0.70573	0.40747	143.33	0.44	0.99	0.38
116	IC156	33.48	0.72043	0.53047	0.56137	0.50508	150.58	0.5	0.96	0.41
117	IC156	44.87	0.78181	0.846	0.41386	0.31959	153.08	0.4	0.98	0.29
118	IC154	40.96	0.78662	0.6875	0.34979	0.40747	162.08	0.6	0.97	0.53
119	IC154	44.43	0.76232	0.66329	0.31863	0.47355	156.73	0.5	0.94	0.49
120	IC154	35.1	0.76467	0.8453	0.22784	0.37208	157.6	0.53	0.96	0.49

121	IC183	75.73	0.53057	0.77367	0.18877	0.37845	163.13	0.82	1	0.2
122	IC183	89.24	0.55917	0.87651	0.16518	0.31331	159.07	0.78	0.97	0.23
123	IC183	89.94	0.39704	0.78074	0.15067	0.14599	157.32	0.86	0.99	0.24
124	IC049	51.57	0.87619	0.94172	0.84782	1.16939	152.95	0.43	0.91	0.2
125	IC049	65.02	0.8041	0.96043	0.89056	1.14856	146.53	0.39	0.91	0.05
126	IC049	59.12	0.92007	0.83627	1.00791	1.62955	147.9	0.4	0.9	0.09
127	IC064	39.53	0.7999	0.57956	0.47534	0.3529	157.86	0.46	0.91	0.41
128	IC064	36.1	0.71801	0.79086	0.5993	0.28837	161.64	0.46	0.85	0.32
129	IC064	39.32	0.93515	0.46083	0.62528	0.3619	176.15	0.371	0.844	0.15
130	IC125	45.3	0.80684	0.90342	1.08686	1.51911	149.25	0.3	0.72	0.04
131	IC125	48.89	0.76184	0.92595	1.05403	1.63075	163.24	0.57	0.94	0.07
132	IC125	45.58	0.76059	0.80611	0.87875	1.45113	172.68	0.31	0.73	0.03
133	IC124	45.11	0.72113	0.66504	0.596	0.36245	159.67	0.59	0.96	0.27
134	IC124	38.56	0.79331	0.68335	0.72068	0.34855	163.81	0.38	0.91	0.23
135	IC124	51.86	0.74751	0.90256	0.92484	1.39596	174.12	0.48	0.9	0.04
136	IC128	58.78	0.83345	0.58625	0.73535	0.28547	154.64	0.43	1	0.25
137	IC128	47.46	0.74257	0.78327	0.86392	1.53295	180	0.31	0.81	0.07
138	IC128	76.94	0.94874	0.88435	0.85578	1.9054	180	0.36	0.98	0.08
139	IC065	55.37	0.58048	0.89413	0.97958	1.8377	151.56	0.36	0.73	0.04
140	IC065	55.11	0.67936	0.83803	0.90949	1.82544	154.86	0.57	0.84	0.29
141	IC065	43.95	0.70039	0.69267	0.89422	1.30377	138.99	0.46	0.83	0.15
142	IC057	51.39	0.82409	0.81319	0.42905	0.48142	164.17	0.45	1	0.53
143	IC057	44.83	0.7669	0.79277	0.35566	0.43431	157.88	0.54	0.97	0.59
144	IC057	42.25	0.76189	0.77964	0.32939	0.47243	150.17	0.5	0.94	0.63
145	IC077	43.73	0.65287	0.70638	0.29168	0.96387	148.06	0.606	0.926	0.378
146	IC077	53.36	0.51031	0.68872	0.27374	0.616	143.93	0.79	0.97	0.48
147	IC077	48.72	0.59216	0.72865	0.27155	0.86843	152.31	0.68	0.95	0.51
148	IC191	50.93	0.83982	1.28706	0.52855	1.59956	199	0.43	0.9	0.06
149	IC191	45.88	0.62079	1.31004	0.51053	1.64444	166.59	0.58	0.92	0.06
150	IC191	49.9	0.65417	1.405	0.57023	1.83388	160.78	0.6	0.96	0.17
151	IC020	37.53	0.76179	0.68612	0.66718	0.63762	161.93	0.55	0.99	0.4
152	IC020	55.85	0.7778	0.61916	0.59456	0.34306	143.73	0.54	0.94	0.46
153	IC020	51.19	0.76773	0.6556	0.59803	0.65032	139.87	0.52	0.95	0.34
154	IC185	38.14	0.63539	1.14876	1.011	1.85416	168.55	0.41	0.86	0.02
155	IC185	51.02	0.74027	0.9448	1.1012	2.51454	170.62	0.323	0.9	0.05
156	IC185	43.73	0.69433	0.94483	0.98001	2.17346	168.13	0.3	0.75	0.01
157	IC043	49.26	0.82521	0.57694	0.70654	0.7432	152.06	0.49	0.89	0.61
158	IC043	50.88	0.78498	0.60417	0.4203	0.2217	161.24	0.44	0.84	0.31
159	IC043	45.38	0.77633	0.63486	0.66505	0.513	130.75	0.52	0.86	0.54
160	IC139	18.89	0.6936	1.03038	0.61543	1.22107	153.69	0.55	0.97	0.35
161	IC139	20.83	0.68833	1.08205	0.46616	1.14703	162.85	0.61	0.95	0.3
162	IC139	38.16	0.69844	1.11232	0.51464	1.80085	173.28	0.62	0.99	0.21
163	IC149	65.27	0.89704	0.30121	0.86063	0.22476	177.96	0.35	1	0.02
164	IC149	63.09	0.9366	0.31003	0.86912	0.33207	174.01	0.34	1	0.06
165	IC149	62.1	0.73221	0.38068	0.92428	0.36812	173.91	0.5	1	0.3
166	IC134	50.23	0.88931	0.37468	0.94155	0.26817	176.13	0.33	1	0
167	IC134	48.66	0.95828	0.26531	0.95352	0.1679	178.78	0.28	1	0.11
168	IC134	49.59	0.89857	0.41319	0.89117	0.37326	163.76	0.51	1	0.06
169	IC265	64.14	0.8561	0.60555	0.47863	0.34674	161.81	0.4	0.85	0.31
170	IC265	57.31	0.91345	0.71925	0.5541	0.35317	159.94	0.498	0.967	0.15
171	IC265	72.12	0.70618	0.67707	0.49498	0.43372	165.75	0.6	0.98	0.33
172	IC018	40.25	0.74634	0.81441	0.32703	0.36298	162.89	0.5	0.96	0.46
173	IC018	41.85	0.71326	0.71231	0.38611	0.45161	152.2	0.61	0.91	0.51
174	IC018	52.64	0.7027	0.71979	0.29691	0.34062	162.23	0.68	0.98	0.43
175	IC129	52.81	0.69438	0.82579	0.52901	0.87559	151.99	0.66	0.98	0.54
176	IC129	60.13	0.65758	0.91452	0.6212	0.99717	150.78	0.65	0.97	0.45
177	IC129	54.67	0.65868	0.90305	0.68888	0.80702	142.18	0.575	0.932	0.457
178	IC168	24.13	0.55342	1.1807	0.41827	1.18808	173.56	0.63	0.98	0.17
179	IC168	29.52	0.85493	0.99604	0.56825	1.17004	177.3	0.54	0.96	0.04
180	IC168	35.13	0.74163	1.06872	0.71482	1.5676	178.33	0.57	0.92	0.2
181	IC013	48.59	0.71599	0.64231	0.42326	0.40296	151.65	0.59	0.89	0.44

182	IC013	50.06	0.63064	0.63823	0.57465	0.46964	154.45	0.56	0.94	0.29
183	IC013	50.41	0.76354	0.45447	0.59319	0.54949	162.84	0.48	0.94	0.51
184	IC005	78.39	0.76132	0.60735	0.44654	0.33742	147.22	0.64	0.96	0.41
185	IC005	80.24	0.77991	0.79275	0.36126	0.40553	166.33	0.58	0.96	0.46
186	IC005	55.65	0.74394	0.79317	0.41097	0.41545	149.57	0.59	0.91	0.46
187	IC117	50.28	0.80549	0.65135	0.58198	0.61555	143.46	0.61	0.94	0.43
188	IC117	51.16	0.77756	0.67416	0.41461	0.52424	146.29	0.59	0.92	0.45
189	IC117	59.43	0.72219	0.69477	0.42044	0.51708	153.91	0.58	0.93	0.48
190	IC131	25.99	0.88543	1.05223	0.82532	0.9336	165.49	0.39	0.88	0.08
191	IC131	30.66	0.77807	1.00674	0.94201	1.14746	170.37	0.51	0.92	0.03
192	IC131	29.98	0.79607	0.89917	0.75556	1.13218	163.93	0.5	0.92	0.05
193	IC121	74.67	0.73939	0.82135	0.06489	0.36855	178.75	0.55	0.97	0.2
194	IC121	68.59	0.74515	0.7227	0.11539	0.35165	167.6	0.58	0.99	0.17
195	IC121	57.89	0.52143	1.00451	0.03662	0.39094	166.66	0.69	0.97	0.13
196	IC267	78.08	0.97272	0.2322	0.88748	0.33069	172.65	0.32	0.96	0.03
197	IC267	32.19	0.94595	0.40447	0.77343	0.379	169.23	0.48	1	0.05
198	IC267	56.51	0.90833	0.33375	0.63362	0.34755	179.02	0.35	1	0.32
199	IC146	43.45	0.80736	0.42509	0.85057	0.34177	188	0.43	1	0.27
200	IC146	46.83	0.86184	0.39035	0.69967	0.27781	179	0.45	0.99	0.21
201	IC146	44.85	0.94181	0.32977	0.61325	0.19554	192	0.23	1	0.45
202	IC122	83.37	0.44989	1.09798	0.06225	0.63677	147.22	0.82	0.95	0.28
203	IC122	90.63	0.47733	0.85899	0.0772	0.18658	150.19	0.81	0.96	0.42
204	IC122	106.7	0.44105	0.82146	0.10143	0.35923	143.09	0.84	0.98	0.25
205	IC017	104.58	0.45477	0.75483	0.10869	0.14726	151.63	0.87	0.98	0.25
206	IC017	98.15	0.47234	0.85196	0.11983	0.13021	150.52	0.8	0.95	0.14
207	IC017	78.37	0.43307	0.90519	0.13462	0.319	147.06	0.79	0.94	0.28
208	IC177	90.52	0.55689	0.82954	0.0815	0.26326	157	0.71	0.93	0.14
209	IC177	101.51	0.52832	0.83263	0.05348	0.18501	141.78	0.78	0.95	0.25
210	IC177	87.76	0.39266	0.93961	0.03626	0.4265	144.23	0.8	0.95	0.24
211	IC015	87.04	0.25597	0.57916	0.07717	0.19278	147.01	0.8	0.97	0.19
212	IC015	81.63	0.17824	0.67585	0.12072	0.40341	160.63	0.89	0.98	0.09
213	IC015	85.55	0.27317	0.62899	0.13269	0.1827	151.9	0.84	0.99	0.31
214	IC080	94.08	0.40083	0.60247	0.06281	0.20111	167.56	0.84	0.97	0.31
215	IC080	71.95	0.24183	0.68228	0.10837	0.24406	156.8	0.8	0.96	0.27
216	IC080	73.46	0.263	0.73564	0.08031	0.25116	171.04	0.87	0.99	0.15
217	IC074	44.03	0.23348	0.71792	0.14616	0.40563	120.03	0.87	0.99	0.05
218	IC074	30.36	0.24517	1.02811	0.05105	0.52557	130.04	0.82	0.98	0.18
219	IC074	40.19	0.39537	0.91739	0.13588	0.31799	140.9	0.82	0.99	0.06
220	IC085	23.18	0.24486	1.10856	0.19672	1.10282	127.2	0.81	1	0.06
221	IC085	31.43	0.34198	1.30704	0.14783	1.66384	144	0.73	0.97	0.09
222	IC085	23.67	0.3516	1.05481	0.23109	0.89216	148.66	0.8	0.98	0.03
223	IC102	46.08	0.30777	1.13274	0.12934	0.76475	135.31	0.85	0.99	0.04
224	IC102	47.81	0.37542	1.13455	0.14997	1.0935	142.23	0.84	0.99	0.05
225	IC102	53.64	0.27082	1.14566	0.13348	1.04314	142.28	0.86	0.99	0.04
226	IC067	32.38	0.37285	1.23824	0.17851	1.01262	123.39	0.78	0.93	0.17
227	IC067	35.54	0.25979	1.42046	0.07428	1.01119	126.15	0.79	0.95	0.07
228	IC067	31.06	0.46787	1.1213	0.18352	1.03249	118.99	0.72	0.92	0.01
229	IC052	52.32	0.28918	0.82263	0.19842	0.36181	149.32	0.88	1	0.28
230	IC052	38.68	0.32704	0.92735	0.27405	0.83661	142.03	0.83	0.99	0.17
231	IC052	43.21	0.38926	0.77852	0.22117	0.50914	140.28	0.83	0.99	0.17
232	IC079	30.59	0.43053	0.97221	0.08272	0.983	149.33	0.79	0.97	0.13
233	IC079	29.44	0.38048	1.03335	0.18444	0.97648	147.12	0.79	0.98	0.21
234	IC079	31.86	0.54269	0.86817	0.16775	0.80038	148.26	0.76	0.94	0.32
235	IC104	48.58	0.39727	0.97623	0.29219	1.00413	137.98	0.75	0.98	0.02
236	IC104	52.44	0.33638	0.80625	0.29376	0.78738	129.78	0.8	0.97	0.02
237	IC104	56.68	0.29375	0.91673	0.22748	0.81881	133.58	0.77	0.96	0.1
238	IC099	48.33	0.27315	1.06033	0.32671	0.88372	146.28	0.87	1	0.04
239	IC099	48.21	0.22486	1.05147	0.26094	0.96227	128.03	0.84	0.99	0.06
240	IC099	52.68	0.34662	0.94021	0.14294	0.72893	142.08	0.86	0.99	0.24
